# Disruption of mitochondrial and lysosomal functions by human *CACNA1C* variants expressed in HEK 293 and CHO cells

**DOI:** 10.3389/fnmol.2023.1209760

**Published:** 2023-06-28

**Authors:** Miriam Kessi, Baiyu Chen, Langui Pan, Li Yang, Lifen Yang, Jing Peng, Fang He, Fei Yin

**Affiliations:** ^1^Department of Pediatrics, Xiangya Hospital, Central South University, Changsha, China; ^2^Hunan Intellectual and Developmental Disabilities Research Center, Changsha, China; ^3^Clinical Research Center for Children Neurodevelopmental Disabilities of Hunan Province, Xiangya Hospital, Central South University, Changsha, China

**Keywords:** *CACNA1C*, molecular mechanisms, mitochondrial dysfunction, lysosomal dysfunction, mitochondrial fusion, mitochondrial fission, mitophagy, loss of calcium current density

## Abstract

**Objective:**

To investigate the pathogenesis of three novel *de novo CACNA1C* variants (p.E411D, p.V622G, and p.A272V) in causing neurodevelopmental disorders and arrhythmia.

**Methods:**

Several molecular experiments were carried out on transfected human embryonic kidney 293 (HEK 293) and Chinese hamster ovary (CHO) cells to explore the effects of p.E411D, p.V622G, and p.A272V variants on electrophysiology, mitochondrial and lysosomal functions. Electrophysiological studies, RT-qPCR, western blot, apoptosis assay, mito-tracker fluorescence intensity, lyso-tracker fluorescence intensity, mitochondrial calcium concentration test, and cell viability assay were performed. Besides, reactive oxygen species (ROS) levels, ATP levels, mitochondrial copy numbers, mitochondrial complex I, II, and cytochrome c functions were measured.

**Results:**

The p.E411D variant was found in a patient with attention deficit-hyperactive disorder (ADHD), and moderate intellectual disability (ID). This mutant demonstrated reduced calcium current density, mRNA, and protein expression, and it was localized in the nucleus, cytoplasm, lysosome, and mitochondria. It exhibited an accelerated apoptosis rate, impaired autophagy, and mitophagy. It also demonstrated compromised mitochondrial cytochrome c oxidase, complex I, and II enzymes, abnormal mitochondrial copy numbers, low ATP levels, abnormal mitochondria fluorescence intensity, impaired mitochondrial fusion and fission, and elevated mitochondrial calcium ions. The p.V622G variant was identified in a patient who presented with West syndrome and moderate global developmental delay. The p.A272V variant was found in a patient who presented with epilepsy and mild ID. Both mutants (p.V622G and p.A272V) exhibited reduced calcium current densities, decreased mRNA and protein expressions, and they were localized in the nucleus, cytoplasm, lysosome, and mitochondria. They exhibited accelerated apoptosis and proliferation rates, impaired autophagy, and mitophagy. They also exhibited abnormal mitochondrial cytochrome c oxidase, complex I and II enzymes, abnormal mitochondrial copy numbers, low ATP, high ROS levels, abnormal mitochondria fluorescence intensity, impaired mitochondrial fusion and fission, as well as elevated mitochondrial calcium ions.

**Conclusion:**

The p.E411D, p.V622G and p.A272V mutations of human *CACNA1C* reduce the expression level of *CACNA1C* proteins, and impair mitochondrial and lysosomal functions. These effects induced by *CACNA1C* variants may contribute to the pathogenesis of *CACNA1C*-related disorders.

## Introduction

Calcium Voltage-Gated Channel Subunit Alpha1 C (*CACNA1C*) encodes an alpha-1 subunit of a voltage-dependent calcium channel (Cav1.2 or L-type calcium channel) (Herold et al., 2023). Cav1.2 channels are distributed widely in the brain, heart, and smooth muscles (Zamponi et al., 2015). *CACNA1C* variants can lead to several conditions including Timothy syndrome which is characterized by neurodevelopmental problems, cardiac arrhythmias, and craniofacial deformities (Herold et al., 2023). The impact of the Cav1.2 channel gating changes (GOF) or loss-of-function (LOF) correlates well with cardiac arrhythmias (Herold et al., 2023). Nevertheless, the same Cav1.2 channel gating changes usually do not correlate with *CACNA1C-*related neurodevelopmental disorders (Herold et al., 2023). Both LOF and GOF *CACNA1C* variants have been detected in patients diagnosed with early infantile epileptic encephalopathy (EIEE), intellectual disability (ID)/global developmental delay (GDD), autism spectrum disorder (ASD), and attention deficit-hyperactive disorder (ADHD) based on recent reviews (Moon et al., 2018; Kessi et al., 2021; Herold et al., 2023). Calcium channel blockers have been shown to be effective for the management of cardiac conditions but not for neurodevelopmental conditions, signifying the possibility of the presence of other unknown mechanisms. Therefore, to rely on the patch clamp recording alone to study the mechanisms of the *CACNA1C*–related neurodevelopment disorders is not adequate.

Based on our recent review on calcium channelopathies, we found one patient who harbored *CACNA1C* GOF variant (Kessi et al., 2021). The patient’s clinical manifestations included epilepsy, GDD, arrhythmias, microcephaly, short stature, lower limb weakness, lower limb atrophy, hyperreflexia, spastic diplegia, multiple dental caries and rhabdomyolysis (Hennessey et al., 2014). The muscle biopsy of the patient unveiled the reduction of the mitochondrial complex I and III enzyme activities (Hennessey et al., 2014). TS2-neo mouse model of Timothy syndrome carrying a GOF variant (G406R) exhibited a reduction in mitochondrial metabolism (Calorio et al., 2019). Mitochondrial-calcium ions homeostasis is very crucial for the generation of adenosine triphosphate (ATP), which helps in neuronal synaptic transmission, synaptic plasticity, learning, and memory (Matuz-Mares et al., 2022). However, mitochondrial calcium ions overload can stimulate the opening of the mitochondrial permeability transition pore (mPTP) resulting in the activation of apoptosis (Matuz-Mares et al., 2022). The highest production of the reactive oxygen species (ROS) in the cells occurs in the mitochondria (Matuz-Mares et al., 2022). In addition to mitochondria, calcium ions regulate diverse lysosomal functions including autophagy (Pereira et al., 2012; Medina and Ballabio, 2015; García-Rúa et al., 2016) and oxidative stress sensing (Li et al., 2012). There is some evidence to support the fact that calcium ions overload or deficiency in neurons can result in neurodevelopmental disorders. It has been shown that too much influx of calcium ions to the neurons plays a major role in the occurrence of Alzheimer’s disease (Guan et al., 2021), Parkinson’s disease (Surmeier et al., 2017), Huntington’s disease, glaucoma, epilepsy, and schizophrenia (Wojda et al., 2008). Whereas, deficient calcium ions entry to the neurons due to the loss of the calcium channel function can lead to episodic ataxia type 2 (EA2) (Brini et al., 2014). A few studies provide some clues that mitochondria might play a role in the occurrence of *CACNA1C*-related neurodevelopmental disorders (Cui et al., 2012; Michels et al., 2018; Calorio et al., 2019; Westhoff and Dixon, 2021).

Currently, most of the available molecular studies on *CACNA1C* variants were based more on the heart-related conditions rather than neurodevelopmental disorders. Therefore, this study aimed to explore the molecular mechanisms of three *CACNA1C* variants in causing neurodevelopmental disorders. We performed several experiments in Human Embryonic Kidney 293 (HEK 293) and Chinese Hamster Ovary (CHO) to explore the role of mitochondria and lysosomes in the pathogenesis of the *CACNA1C*-related neurodevelopmental disorders. We show that both mitochondrial and lysosomal dysfunctions are involved in the pathogenesis of this channelopathy. In addition, this study highlights that *CACNA1C* pathogenic variants can be co-localized in the nucleus, mitochondria, and lysosomes. Other previous studies explored the role of mitochondria alone in the mechanisms. Our novel findings may assist in understanding the molecular mechanisms of *CACNA1C*-related neurodevelopmental disorders, and pave the way for the identification of potential treatment targets.

## Materials and methods

### Ethical clearance and patients

The study complies with all ethical regulations and was approved by the Institutional Ethics Committee of the Xiangya Hospital, Central South University. The written informed consents were obtained from the patients, parents, or guardians. We recruited pediatric patients diagnosed with *CACNA1C*-related neurodevelopmental disorders at Xiangya Hospital, Central South University from July 2019 to December 2021. All patients were evaluated by at least two pediatric neurologists, and comprehensive clinical data were collected. Genetic results were also collected and analyzed by the geneticist.

### Genetic sequencing, mutations analysis, and interpretation

Blood samples of the patients and their biological parents were collected, and the genomic DNAs were extracted. The exome library preparation, sequencing, bioinformatics filtering, and data analyses were conducted based on the previous protocols. Whole exome sequencing (WES) was carried out for all three patients. Sanger sequencing was used to verify the parenteral origin of the identified variants. The genetic results were collected and interpreted according to the variant curation guidelines published in 2015 by the American College of Medical Genetics (ACMG) (Richards et al., 2015).

### Cell culture, plasmid construction, and transfection

HEK 293 and CHO cells were purchased from Shanghai-Cell Bank ATCC, and were cultured in Dulbecco’s modified Eagle’s medium (DMEM) basic, and DMEM/F12 (Thermo Fisher Scientific), respectively. Both DMEM and DMEM/F12 were supplemented with 5% inactivated fetal bovine serum (Thermo Fisher Scientific), and 1% penicillin–streptomycin. Both cell models were cultured in 5% CO2, and 37°C. The pcDNA3.1-t2A-EGFP2 was constructed for wild type (WT) and mutants. The WT, p.E411D, p.V622G, and p.A272V were constructed at TsingKe Biotechnology. The other Cav2.1 calcium channel subunits (β3 + α2/δ1) were gifts from the Hunan Normal University. Plasmids were transfected into HEK 293 and CHO cells by using Lipofectamine 2000 (Invitrogen; Thermo Fisher Scientific Inc.) according to the manufacturer’s instructions. All experiments were performed at or after 48 h of transfection. CHO cells were used for mito-tracker and lyso-tracker fluorescence studies only.

### Electrophysiological studies

HEK 293 cells were transfected with Cav1.2 channel subunits (α1A + β3 + α2/δ1) by using 1.6 μg of each subunit plasmid in a ratio of 1:1:1 as per previous protocol (Jiang et al., 2019). The whole-cell electrophysiological studies were done by using the Multiclamp 700B amplifier at room temperature (20–22°C). The extracellular solution was made by the mixture of barium chloride [20 millimolar (mM)], sodium chloride (100 mM), magnesium chloride (1 mM), tetraethyl ammonium chloride (20 mM), *N*-2-hydroxyethylpiperazine-N-2-ethane sulfonic acid (HEPES 10 mM) and glucose (10 mM), at 7.3 pH and the osmolality of about 300 milliosmolar (mOsm). The intracellular solution was made up of a mixture of cesium chloride (120 mM), magnesium chloride (1 mM), ethylene glycol tetraacetic acid (5 mM), HEPES (10 mM), magnesium-ATP (5 mM) and GTP disodium salt (0.3 mM), at 7.3 pH and osmolality of about 275 mOsm. The tip of the glass microelectrode mounted on the microelectrode holder was gradually moved toward the cell surface. The negative pressure was applied when it contacted the envelope, and the electrode tip was gently sucked to form a high impedance sealing of more than 1GΩ with the cell surface for fast capacitance compensation. A small amount of negative pressure was given to break the membrane in order to form a whole-cell recording mode, and voltage-clamp mode was adopted after forming high-impedance sealed cells. The slow capacitance compensation and series resistance compensation were adjusted to reduce the instantaneous charge, discharge current, and clamp error. The leakage current and capacitive current were subtracted by A-P/4 protocol, and the signal was set to be filtered by a low-pass filter with a cutoff frequency of 2 kHz and sampling frequency of 10 kHz.

Cells were clamped at −80 mV, voltage testing was started from −60 mV, and then stimulated to +60 mV in a step of 10 mV, within 140 ms duration. The calcium currents at different voltages were recorded. Current density (pA/pF) = current intensity/membrane capacitance. The current intensity was the ratio of the current during activation to the maximum activation current. The current densities were measured in live cells only. Time constants (τ) of activations were quantified on currents evoked at 10 mV according to the equation (Y = Ai exp. (−t/τ) + C). The voltage-dependence of activations were assessed from the conductance (G)-voltage relationship obtained by the equation G = I/ (Vm − Vrev), where Vrev is the extrapolated reversal potential. All data were acquired by using a 700 B amplifier (Molecular Devices), digitized with the Axon™ Digidata® 1550B plus HumSilencer® (Axon Instruments), and analyzed using Clampfit 10.6 software (Axon Instruments).

### RNA extraction and real-time quantitative PCR

Primers were constructed by using Primer Bank. Primers for the *CACNA1C*, apoptotic genes (*Bax*, *BCl-2*, and *Caspase-3*), autophagy genes (*LAMP1*, *LC3II*, *Beclin-1*, and *p62*), and mitophagy genes (*PINK1* and *PARKIN*) were used in this study (Supplementary Table S1). Ribonucleic acids (RNAs) were extracted according to the E.Z.N.A Total RNA Kit (Omega kit). About 1,000 ng of RNA for each mutant was reverse-transcribed according to the instructions of Hifair II 1st Strand cDNA synthesis super mix for quantitative PCR (qPCR). Real-time quantitative PCR (RT-qPCR) was performed by using SYBR green as a fluorescent dye (Yeasen, China) on ABI 7500 (Applied Biosystems; Thermo Fisher Scientific, Inc.). The relative mRNA expression of the target genes was calculated based on the 2^-ΔΔCT^ method.

### Protein extraction and western blotting

Protein was extracted from the whole cell, cell membrane, and nucleus of the HEK 293 cells. The whole cell protein was extracted with a radio immunoprecipitation assay (RIPA) mixed with phenylmethanesulfonyl fluoride (PMSF). The cytoplasmic protein and nucleoprotein were extracted with a nuclear and cytoplasmic protein extraction kit (cat. no. KGP150/KGP1100; Nanjing KeyGen Biotech. Co., Ltd.). The protein concentration was measured using bicinchoninic acid (BCA) protein assay kit (Pierce). Equal amounts of 35 μg proteins from each sample were loaded to 12.5% sodium dodecyl sulfate (SDS) polyacrylamide gel and separated by electrophoresis (80 V for 60 min, then 120 V for 1–2 h). Then, proteins were transferred to 0.45 μm polyvinylidene difluoride (PVDF) membranes (Millipore) (300 mVA for 105 min). Membranes were blocked with protein-free rapid blocking buffer (product code 20B10) at room temperature for 30 min. Membranes were then incubated with primary antibodies at 4°C overnight (in a shaker machine) to detect the effects of different variants on apoptotic proteins (Bax, BCl-2, Caspase-3, and PARP), autophagy proteins (LAMP1, LC3II, Beclin-1, and p62), mitophagy proteins (PINK1 and PARKIN), mitochondrial fusion and fission proteins (OPA1 and DRP1), mitochondrial enzyme proteins (MT-CO1 and SDHA), and endoplasmic reticulum stress (ERS) protein (DDIT3/CHOP) expression. We used actin as an internal control for all target proteins from the whole lysates, and GAPDH for the cytoplasmic and nuclear proteins.

After washing membranes in phosphate-buffered saline with Tween-20 solution (PBST) three times for 10 min, depending on the origin of the primary antibodies, membranes were incubated with either HRP-conjugated AffiniPure goat anti-mouse secondary antibody or HRP-conjugated AffiniPure goat anti-rabbit secondary antibody at room temperature for 1 h. Supplementary Table S2 provides detailed information on the antibodies used in this paper. The chemiluminescence signals of the target proteins were visualized by using New Super ECL Assay (cat number: KGP1127-KGP1128) in ChemiDoc XRS+ system (Bio-Rad Laboratories, Inc.). Densitometric values of the blots were analyzed by using Image J.

### Apoptosis assay

The transfected cells were stained with the Annexin V-APC/7-AAD (cat number P-CA-208, Procell Life Science & Technology Co., Ltd) for 15 min in the dark and analyzed using a flow cytometer (FACScan; BD Biosciences). The FlowJo analysis software (version 10.0; FlowJo LLC) was used for the final data analysis. The cells in the quadrants represented different cell states as follows: dead cells were presented in the left upper quadrant (Q1), late-apoptotic cells were presented in the upper right quadrant (Q2), viable cells were presented in the lower left quadrant (Q4), and early apoptotic cells were presented in the lower right quadrant (Q3).

### Measurement of intracellular ROS

The Ab113851DCFDA/H2DCFDA – Cellular ROS Assay kit was used to measure the generation of ROS in HEK 293 cells. ROS production was monitored with the ROS-sensitive fluorescent probe 5-amino-2, 3-dihydro-1, 4-phthalazinedione (3-aminophthalhydrazide; luminol, Sigma) according to the manufacturer’s instructions. A microplate reader (Biotek Synergy H1, USA) was used for reading.

### Determination of the ATP production rate

The ATP production rate was determined by the ATP bioluminescence assay kit (CLS II kit). The transfected cells were incubated at 100°C for 2 min with 9 times the volume of boiling 100 mM Tris and 4 mM EDTA solution at pH 7.75. They were then centrifuged at 1000 g for 60 s, the supernatant was transferred to a new centrifuge tube and placed on ice. Then, 50 μ l cell of supernatant/ATP standard was transferred into a white 96 well plate. Thereafter, 50 μl of luciferase working solution was added into each hole containing a sample, and luminosities were measured by the microplate reader (Biotek Synergy H1, USA).

### Determination of mitochondrial complex I activity

The quantification of the complex 1 activity was conducted according to the instructions of the Complex I Enzyme Activity Assay Kit (Colorimetric) (Abcam, cat number, ab109721). Transfected cells were placed over the ice and then washed with PBS twice. Thereafter, the detergent was used to extract proteins. Detergent extract of prepared samples were loaded onto a plate, and then incubated for 3 h at room temperature. The plate wells were thereafter washed with washing buffer three times. Then 200 μl of assay solution of each sample was added to each well. The optical density (OD450 nm) in kinetic mode was measured with a microplate reader (Biotek Synergy H1, USA) at room temperature for up to 30 min.

### Mitochondrial copy numbers

Mitochondrial DNA copy number determination: after genomic DNA extraction according to the E.Z.NA Tissue DNA Kit (Omega BIO-TEK), the relative level of mitochondrial DNA copy number was determined by RT-qPCR according to the SYBR Green fluorescent dye instructions (Yeasen, China). The target genes included mitochondrial (*mt-ND1*, *hMito-1*, *h-16S RNA-1*, and *mt3212*) and nuclear (*B2M*). Supplementary Table S3 lists the primers used for checking mitochondrial copy numbers. Triplicate amplifications of the mitochondrial and nuclear products were carried out. We calculated the ratio of mitochondrial/B2M copy numbers of each sample hole and compared the relative copy number ratios between samples.

### Mito-tracker red staining

Chinese hamster ovary cells were washed with PBS twice after 48 h of transfection. Then cells were stained with mito-tracker red (MitoTracker Red CMXRos, Invitrogen, Thermo Fisher Scientific). Mito-tracker red was added directly to the cell culture medium in the ratio of 1:10000, and incubated for 30 min. The cells were washed with PBS twice and then observed and photographed by using an Inverted fluorescence microscope (ZEISS Technology Co. Ltd) under 63 oil objective. Photographs were analyzed by using Image J.

### Lyso-tracker red staining

Chinese hamster ovary cells were washed with PBS twice after 48 h of transfection. Thereafter, cells were stained with lyso-tracker red (Lyso Tracker Red DND-99 Lot number 2204208, Invitrogen, Thermo Fisher Scientific). Lyso-tracker red was added directly to the cell culture medium in the ratio of 1:10000, and incubated for 30 min. Cells were washed with PBS twice, then observed and photographed by using an Inverted fluorescence microscope (ZEISS Technology Co. Ltd) under 63 oil objective. Photographs were analyzed by using Image J.

### Mitochondrial calcium concentration test

Mitochondrial calcium ion levels were determined by using Rhod-2 AM. The transfected cells were rinsed with Hanks Balanced Salt Solution (HBSS) three times and stained with a mixture of 5 μM Rhod-2 AM diluted in HBSS at 37°C for 5 min in the dark. Finally, live cells were rinsed thrice by adding HBSS buffer, and thereafter incubated at 37°C for 10 min in the dark. Cells were then analyzed by using a flow cytometer (FACScan; BD Biosciences). The FlowJo analysis software (version 10.0; FlowJo LLC) was used for the final data analysis.

### Cell viability assay

Cells were seeded on a 96-well culture plate, 48 h post-transfection. The enhanced cell counting kit-8 (CCK-8; Biosharp Life Sciences) was utilized to assess cell viability according to the manufacturer’s instructions. In brief, 10 μl of the CCK-8 solution was added to each culture hole and incubated for 4 h at 37°C. The absorbance was measured at 450 nm with the microplate reader (Biotek Synergy H1, USA).

### Statistical analysis

The data were entered and processed by the GraphPad Prism 7.0 software. All data were expressed as the mean ± standard deviation. Two independent sample t test was used for comparison between the two groups. *p* < 0.05 indicated statistically significant differences (*p < 0.05 as indicated in the images and figure legends). All experiments were repeated at least three times.

## Results

### Clinical features and genetic results

**Figure 1:**
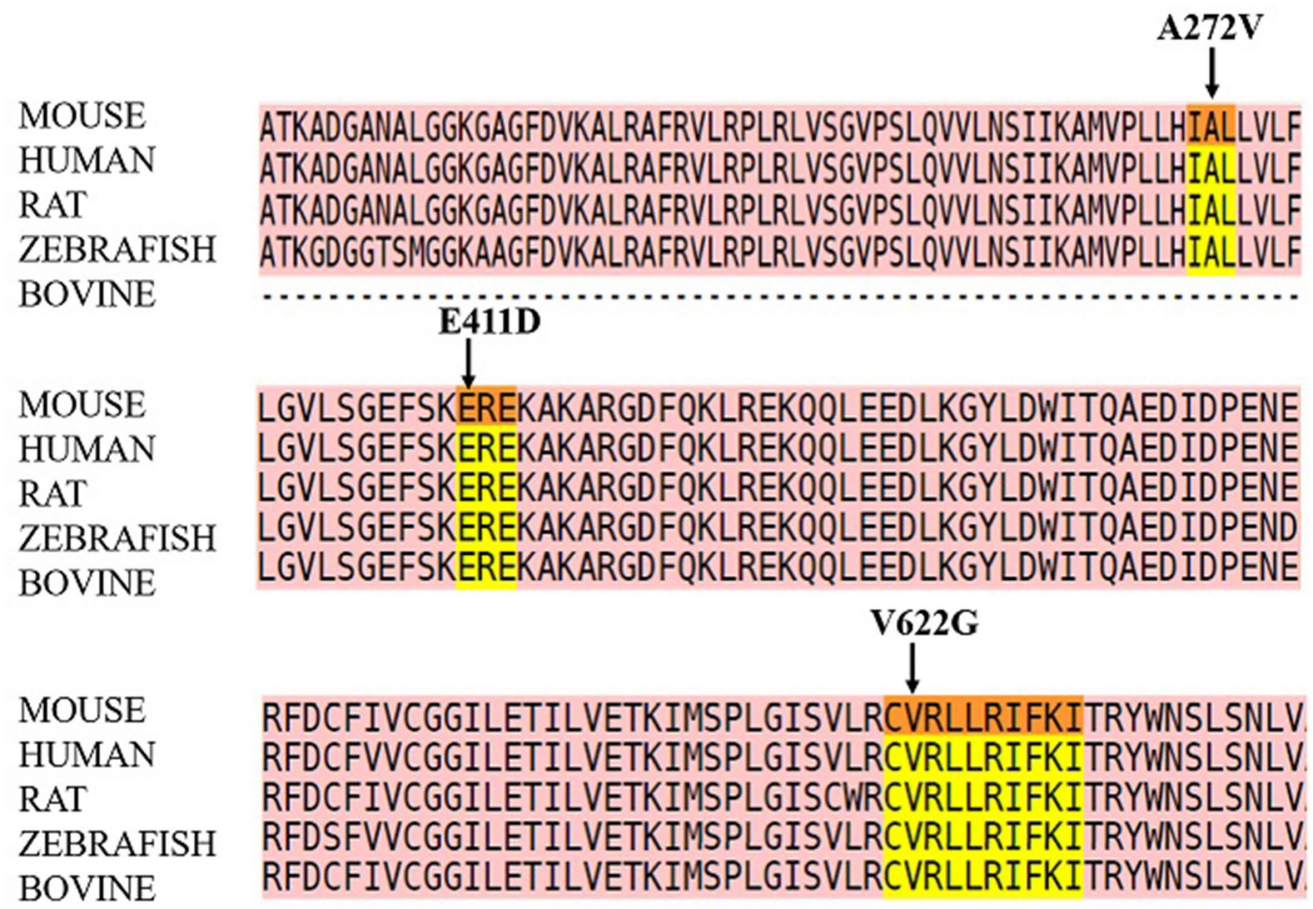
Protein sequence alignment of the three mutants.

Three cases were enrolled in this study. Two females and one male. The first patient was diagnosed with ADHD, sinus arrhythmia, and moderate ID. The second patient was diagnosed with West syndrome, GDD, patent foramen ovale, and arrhythmia. The third patient presented with epilepsy and mild ID (Table 1). These patients carried 3 novel, *de novo CACNA1C* variants: p.E411D located on the cytoplasmic region, p.V622G located on S4 domain II, and p.A272V located on S5 domain I. Two were likely pathogenic variants and one was a variant of unknown significance (VUS) (Supplementary Table S4). All three variants are functionally conserved across different species (Figure 1).

**Table 1:** Clinical characteristics of three patients with *CACNA1C*-related neurodevelopmental disorders in this study.

Patients	P1	P2	P3
Mutation	c.1233G > C: p.E411D located on cytoplasmic region	c.1865 T > G: p.V622G located on S4 domain II	c.815C > T: p.A272V located on S5 domain I
Age/Sex	9y11mo/F	6mo/M	1y2mo/F
Age of seizure onset	No seizures	3 m	9 m
Seizure semiology	NAD	Spasms and focal seizures	Generalized seizures
Seizure frequency	NAD	Convulsions 2–3 times per day	Unknown
Age of seizure control	NAD	Unknown	3y8m
Presence of the status epilepticus	NAD	No	No
History of febrile seizures	NAD	No	Yes
EEG findings	Normal	Multifocal sharp spike waves, slow waves, spike-slow waves during wake-up period and hypsarrhythmia during sleep	Multifocal sharp waves, spike waves, and spike-slow waves during the waking-up period, which were observed bilaterally in the rolandic area.
ECG	Sinus arrhythmia	High arrhythmia during sleep	Normal
Brain MRI/CT scan	Normal	left temporal pole extra cerebral space widened	The right ventricle temporal horn is slightly larger
Treatment strategies		VPA and ACTH	VPA
Severity of ID/GDD	Moderate	Moderate GDD	Mild
Language development	Severe delay		
Family history	No	No	Family history of ID
Other clinical features	No	Kidney stones with hydronephrosis. Bilateral inguinal oblique hernia.	No
Diagnosis	ADHD and moderate ID	WS, GDD and patent foramen ovale	Epilepsy and ID

ACTH, adrenocorticotrophic hormone; ADHD, attention deficit/hyperactive disorder; EEG, electroencephalograph; ECG, electrocardiogram; F, female; GDD, global developmental delay; I, intellectual disability; M, male; Mo, month; MRI, magnetic resonance imaging; NAD, not applicable; VPA, sodium valproate; WS, West syndrome; Y, year.

### Mutants exhibited low current densities, reduced mRNA, and protein expression

**Figure 2:**
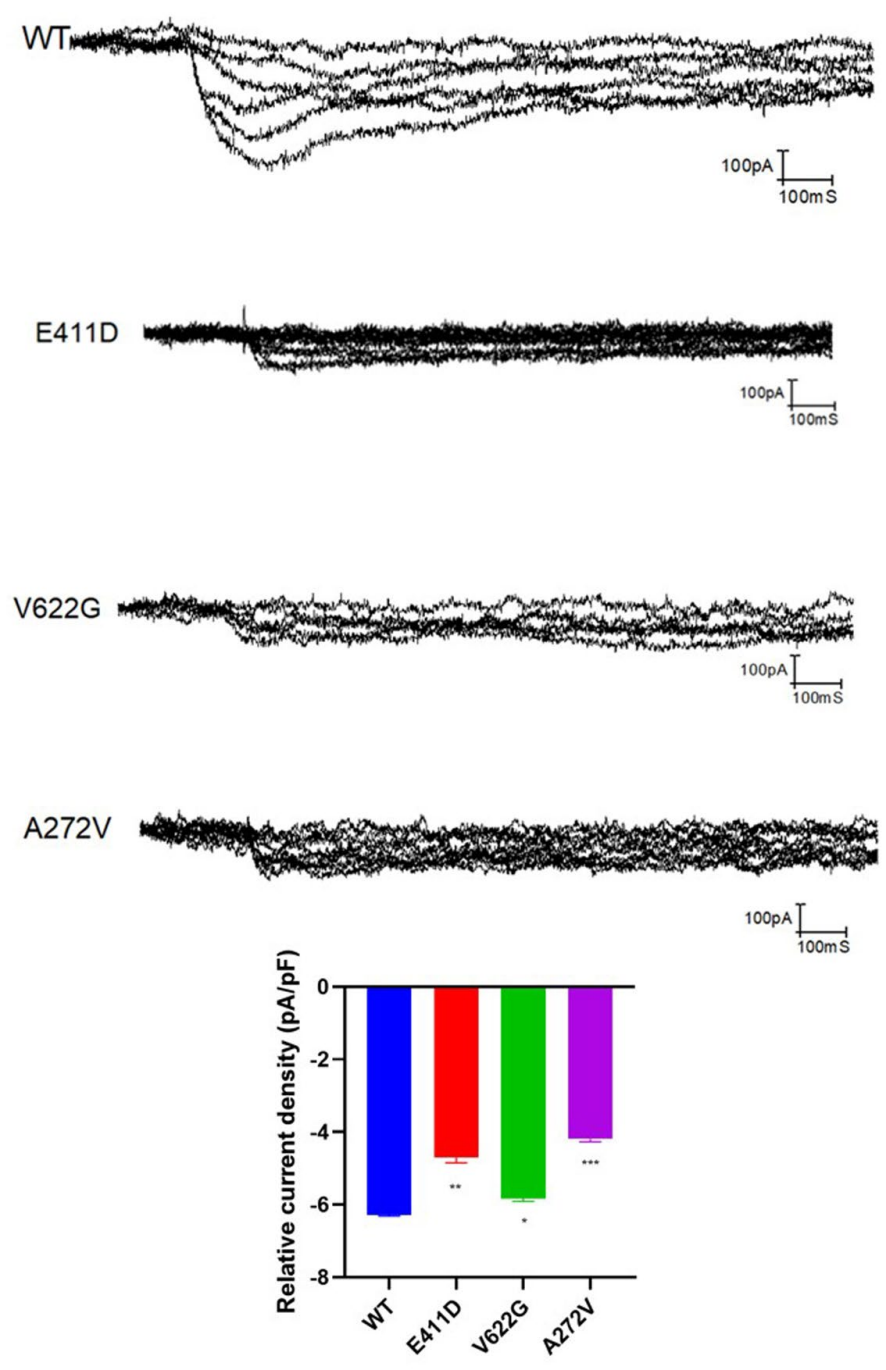
The relative current density of the *CACNA1C* variants based on the electrophysiological studies. Values represent mean ± SEM of more than three independent experiments each. *p < 0.05, **p < 0.01, ***p < 0.001, ****p < 0.0001, NS, non-significant; C, negative control, and WT, wild type.

**Figure 3:**
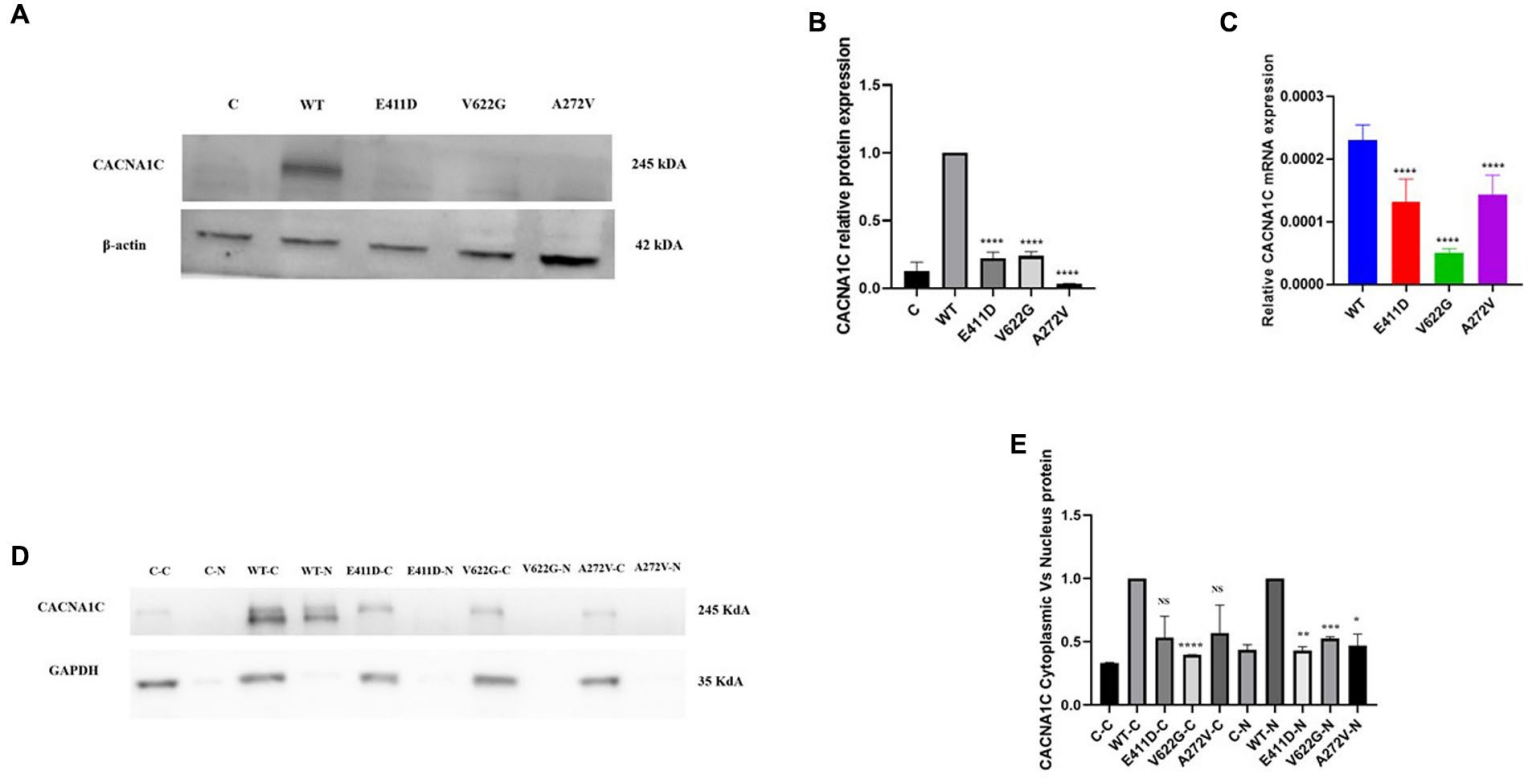
Total, nuclear and cytoplasmic proteins expression levels of the *CACNA1C* variants as well as mRNA expressions. **(A)** Immunoblots of the CACNA1C total protein expression. **(B)** Histogram showing the relative CACNA1C protein values of respective immunoblots. **(C)** Histogram showing relative CACNA1C mRNA expression. **(D)** Immunoblots of the CACNA1C nuclear and cytoplasmic protein expressions. **(E)** Histogram showing the relative CACNA1C nuclear and cytoplasmic proteins values of respective immunoblots. Values represent mean ± SEM of more than three independent experiments each. *p < 0.05, **p < 0.01, ***p < 0.001, ****p < 0.0001, NS, non-significant; C, negative control; WT, wild type; C-C, negative control cytoplasmic protein; C-N, negative control nuclear protein, and N, nuclear.

Electrophysiological studies of the mutants revealed reduced relative calcium current densities for all three variants (Figure 2). All three mutants exhibited considerably lower whole cell protein expression than WT (Figures 3A,B). Likewise, all mutants had lower mRNA expression than WT (Figure 3C). A similar protein trend was observed when cytoplasmic and nuclear proteins were extracted: mutants demonstrated lower cytoplasmic and nuclear proteins than WT (Figures 3D,E).

### Mutants induced apoptosis

**Figure 4:**
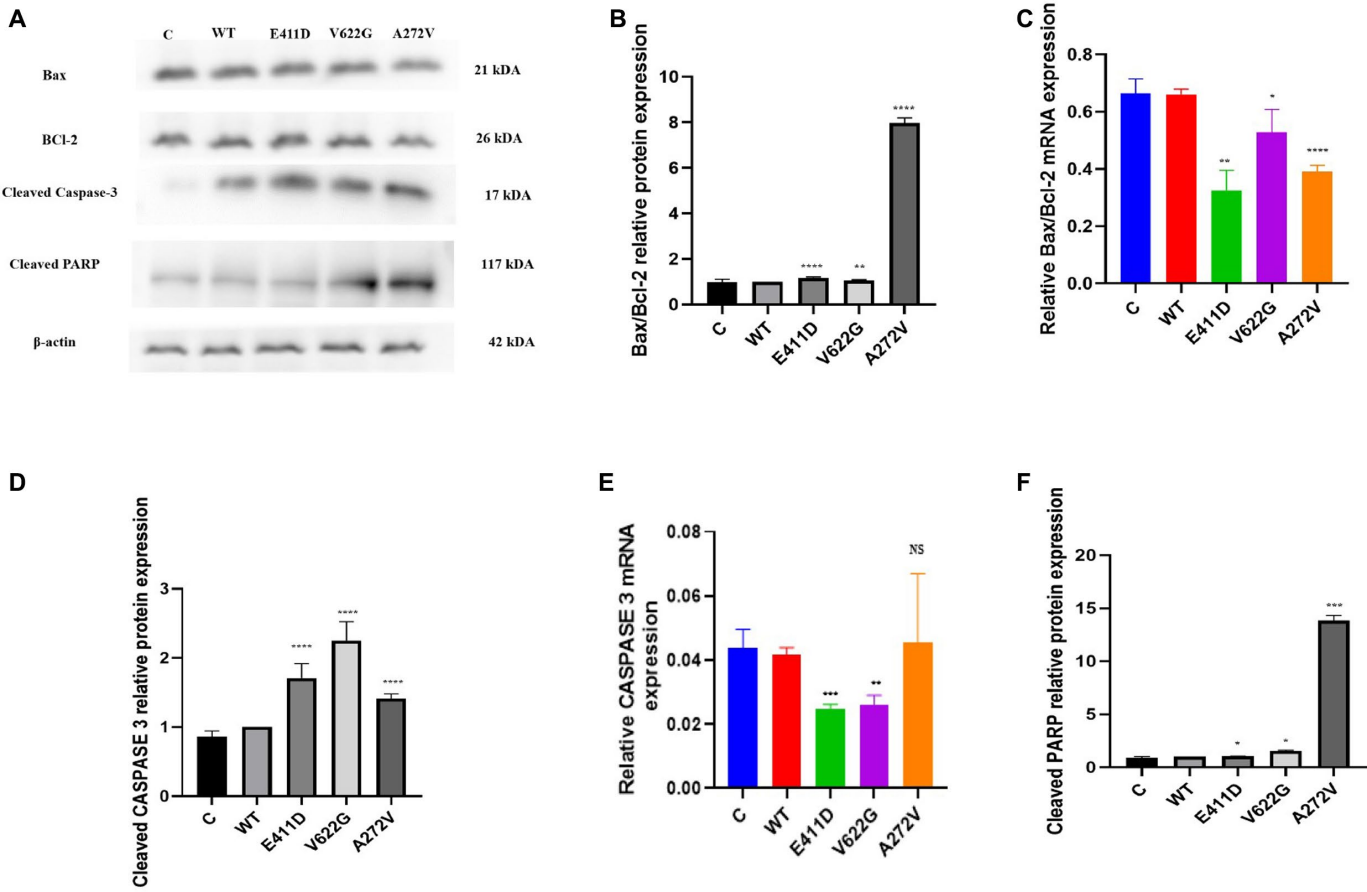
Western blot analysis and qPCR results for the apoptosis markers. **(A)** Immunoblots of the apoptosis protein markers. **(B)** Histogram showing the relative Bax/Bcl-2 protein values of respective immunoblots. **(C)** Histogram showing relative Bax/Bcl-2 mRNA expression. **(D)** Histogram showing the relative cleaved Caspase-3 protein values of respective immunoblots. **(E)** Histogram showing relative caspase-3 mRNA expression. **(F)** Histogram showing the relative cleaved PARP protein values of respective immunoblots. Values represent mean ± SEM of more than three independent experiments each. *p < 0.05, **p < 0.01, ***p < 0.001, ****p < 0.0001, NS, non-significant; C, negative control; and WT, wild type.

**Figure 5:**
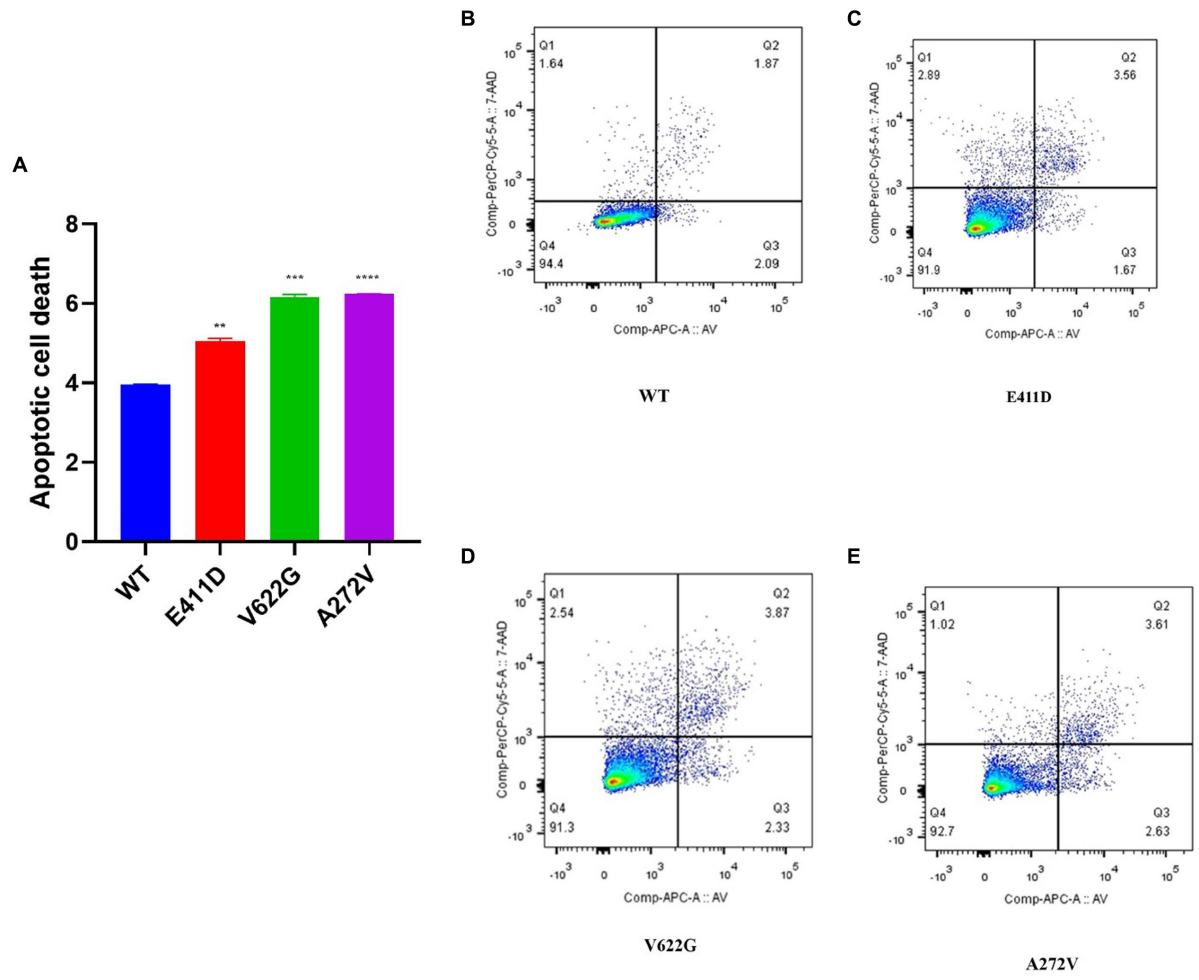
Apoptotic cell death assay results according to the flow cytometry. **(A)** Histogram showing the relative apoptotic cell death. **(B–E)** Apoptosis rates for WT and mutants. Values represent mean ± SEM of more than three independent experiments each. *p < 0.05, **p < 0.01, ***p < 0.001, ****p < 0.0001, NS, non-significant; C, negative control, and WT, wild type.

The mRNAs of the Bax/Bcl-2 for all mutants were considerably lower than WT (Figure 4C). All mutants demonstrated higher Bax/Bcl-2 protein levels than WT (Figures 4A,B). We speculate that high protein expression could impair mRNA expression via a negative feedback mechanism. The p.E411D and p.V622G variants expressed lower mRNA of the Caspase 3 than WT (Figure 4E). All variants expressed considerably higher Cleaved caspase 3 protein than WT (Figures 4A,D). Besides, all mutants expressed significantly higher cleaved PARP protein than WT (Figures 4A,F). Altogether, these results suggested the activation of the intrinsic apoptotic pathway. In addition, all mutants exhibited a significantly higher rate of cell death than WT according to the apoptosis assay. The p.V622G and p.A272V variants induced the highest death rates followed by the p.E411D (Figures 5A–E). It has been shown that calcium homeostasis in mitochondria plays a crucial role in cell physiology and pathophysiology, and is critical in cell death (Matuz-Mares et al., 2022).

### Mutants affected autophagic-lysosomal system

**Figure 6:**
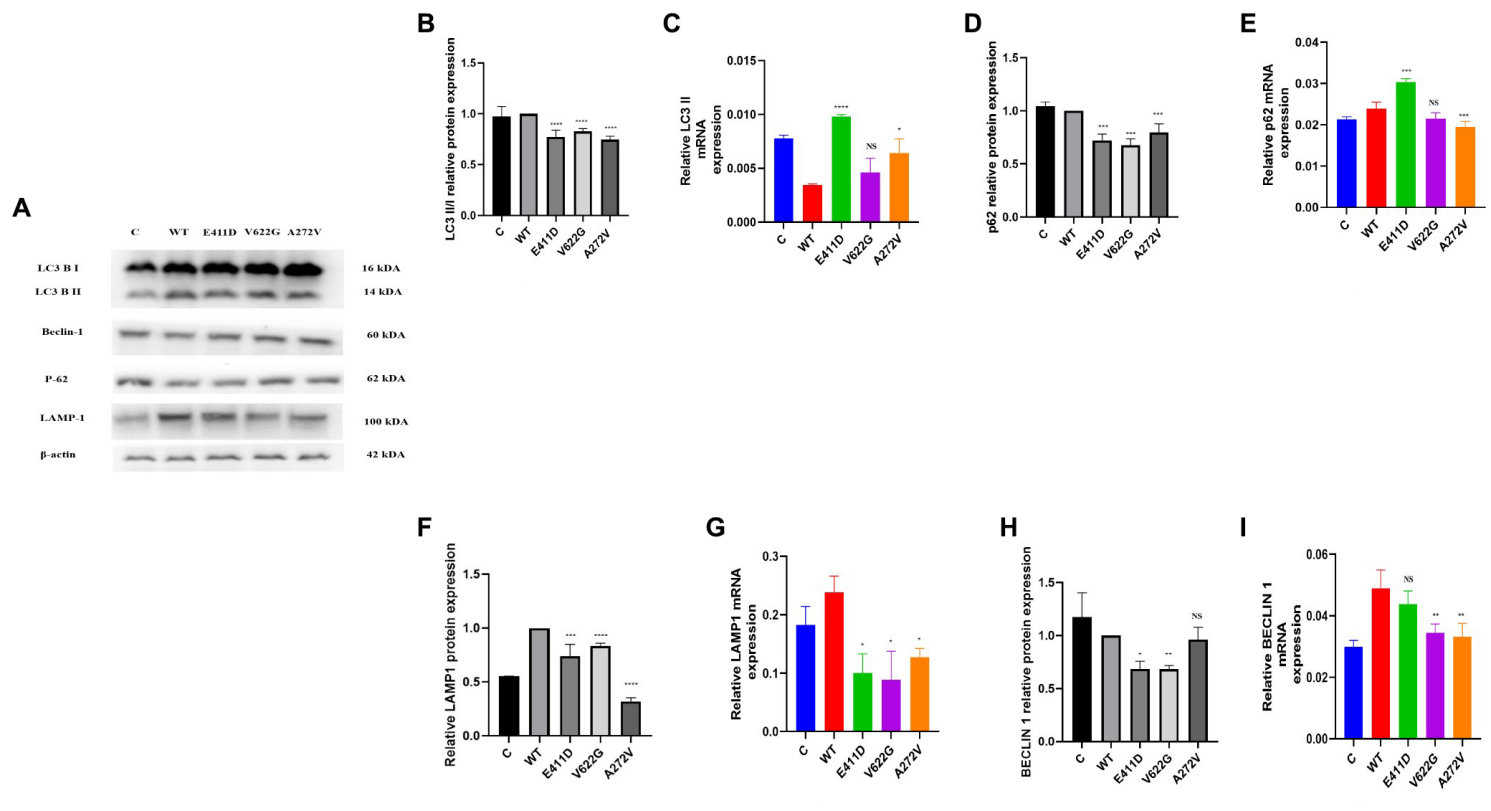
Western blot analysis and qPCR results for the autophagy – lysosomal system. **(A)** Immunoblots of the autophagy – lysosomal system protein markers. **(B)** Histogram showing the relative LC3 II/I protein values of respective immunoblots. **(C)** Histogram showing relative LC3 II mRNA expression. **(D)** Histogram showing the relative p62 protein values of respective immunoblots. **(E)** Histogram showing relative p62 mRNA expression. **(F)** Histogram showing the relative LAMP1 protein values of respective immunoblots. **(G)** Histogram showing relative LAMP1 mRNA expression. **(H)** Histogram showing the relative Beclin-1 protein values of respective immunoblots. **(I)** Histogram showing relative Beclin-1 mRNA expression. Values represent mean ± SEM of more than three independent experiments each. *p < 0.05, **p < 0.01, ***p < 0.001, ****p < 0.0001, NS, non-significant; C, negative control; and WT, wild type.

**Figure 7:**
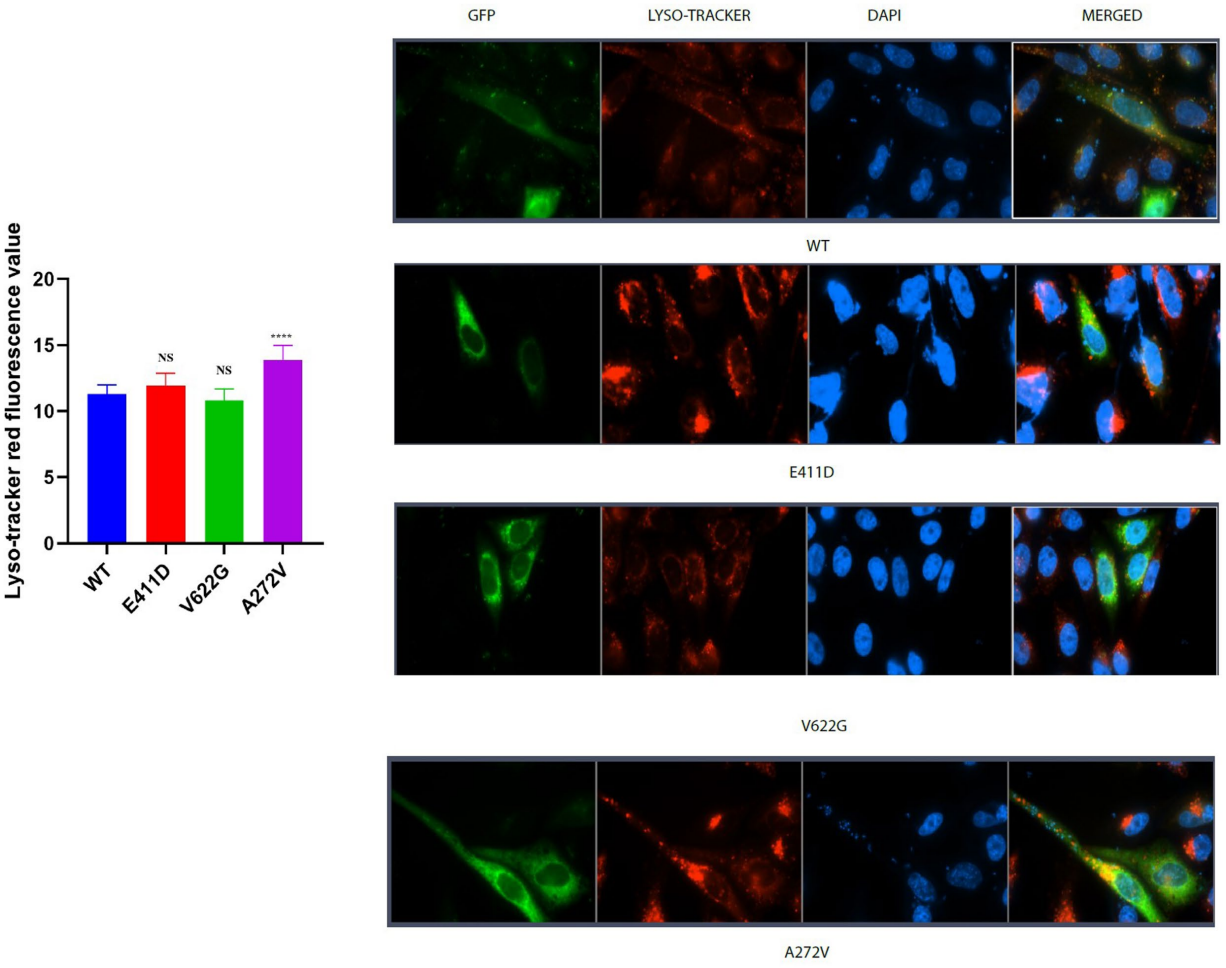
Lyso-tracker red immunofluorescence intensity results. Histogram showing lyso-tracker red fluorescence values. CACNA1C protein is localized in the nucleus, cytoplasm and lysosomes. Values represent mean ± SEM of more than three independent experiments each. *p < 0.05, **p < 0.01, ***p < 0.001, ****p < 0.0001, NS, non-significant; C, negative control; and WT, wild type.

The p.E411D and p.A272V variants demonstrated significantly higher LC3 II mRNA expression than WT (Figure 6C). All three mutants expressed lower LC3II/I proteins than WT (Figures 6A,B). The p.E411D exhibited considerably higher p62 mRNA expression than WT while p.A272V showed significantly lower p62 mRNA expression (Figure 6E). All three variants demonstrated lower p62 protein expression than WT (Figures 6A,D). All three variants expressed lower mRNA and protein levels of LAMP1 than WT, notably, the p.A272V had the lowest protein expression (Figures 6A,F,G). We speculated that low LAMP1 protein expression for the p.A272V occurred as a negative mechanism to the augmented levels of the lysosomes as shown in the section below (increased lysosomes fluorescence intensity). The p.V622G and p.A272V variants demonstrated lower Beclin-1 mRNA expression than WT (Figure 6I). The p.E411D and p.V622G variants exhibited considerably lower Beclin-1 protein expression than WT (Figure 6H). The p.A272V demonstrated significantly higher lysosomes’ fluorescence intensity than WT but there was no significant change for the remaining two mutants. In addition, we could observe that CACNA1C was co-localized with lysosomes (Figure 7).

### Mutants exhibited mitochondrial dysfunctions and impaired mitophagy

**Figure 8:**
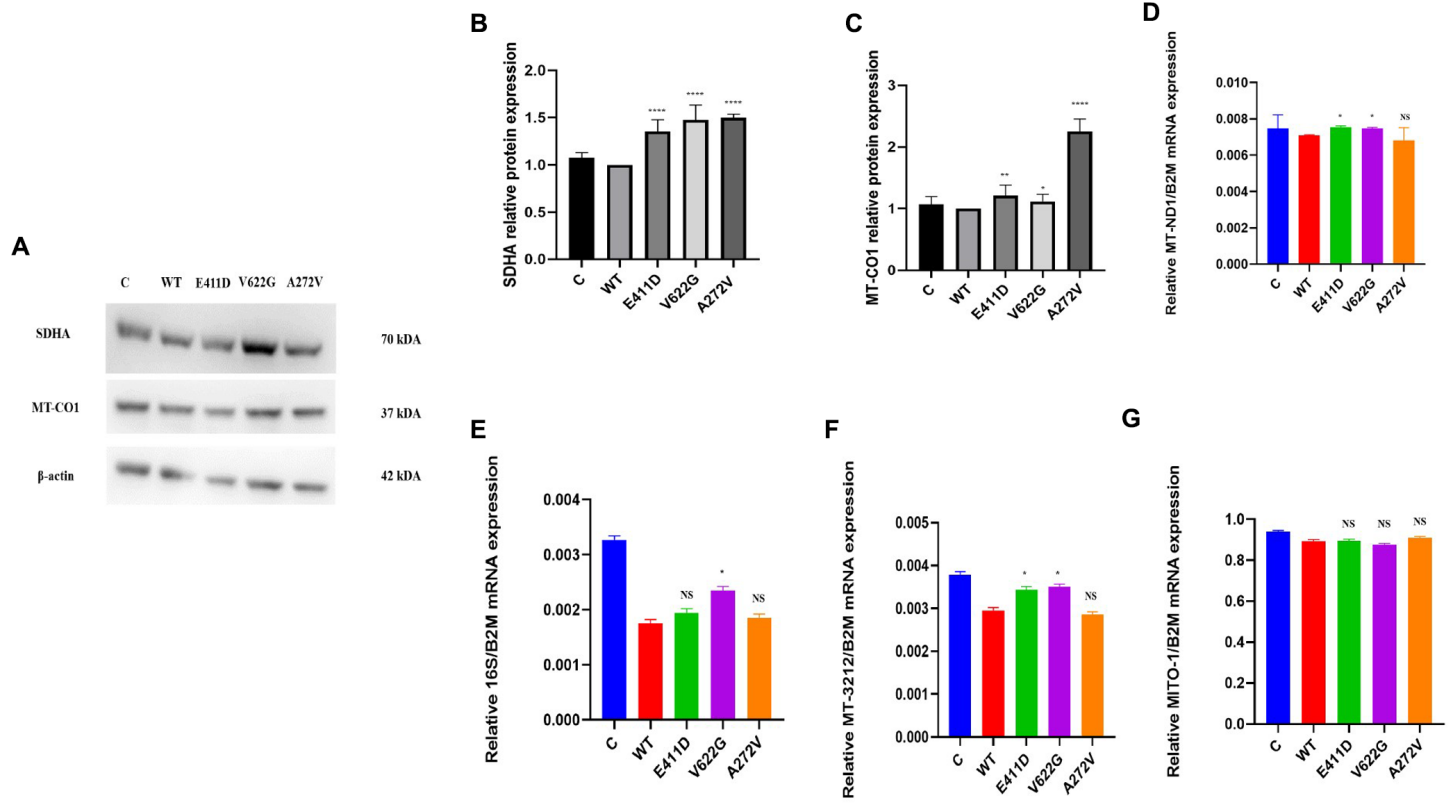
Mitochondrial copy numbers and proteins. **(A)** Immunoblots of the SDHA and MT-CO1 proteins. **(B)** Histogram showing the relative SDHA protein values of respective immunoblots. **(C)** Histogram showing the relative MT-CO1 protein values of respective immunoblots. **(D)** Mitochondrial copy numbers of the MT-ND1/B2M. **(E)** Mitochondrial copy numbers of the 16S/B2M. **(F)** Mitochondrial copy numbers of the MT-3212/B2M. **(G)** Mitochondrial copy numbers of the MITO-1/B2M. Values represent mean ± SEM of more than three independent experiments each. *p < 0.05, **p < 0.01, ***p < 0.001, ****p < 0.0001, NS, non-significant; C, negative control, and WT, wild type.

We checked numerous mitochondrial gene copy numbers and protein levels to see whether there was any dysfunction. Succinate dehydrogenase complex II, subunit A (SDHA) protein is encoded by the *SDHA* gene. All mutants demonstrated higher SDHA protein expression than WT (Figures 8A,B). Mitochondrial Encoded Cytochrome C Oxidase I (MT-CO1) protein was also elevated than WT for all mutants (Figures 8A,C). MT-CO1 encodes for the cytochrome c oxidase, the last enzyme in the mitochondrial electron transport chain which determines oxidative phosphorylation. We also checked the mitochondrial copy numbers. The oxidative stress in human cells can upregulate the expression of the mitochondrial copy numbers as a feedback mechanism that compensates for defects in mitochondria carrying mutated mitochondrial DNA (mtDNA), and a malfunctioning respiratory system (Lee et al., 2000). The p.E411D and p.V622G variants exhibited higher MT-ND1/B2M mitochondrial copy numbers than WT (Figure 8D). The p.V622G variant demonstrated considerably higher h-16 s/B2M mitochondrial copy numbers than WT but there was no change for the rest of the mutants (Figure 8E). The p.E411D and p.V622G variants exhibited higher MT-3212/ B2M mitochondrial copy numbers than WT (Figure 8F). There was no change in the hMITO-1/B2M mitochondrial copy numbers (Figure 8G). Therefore, it seems the mitochondrial copy numbers were elevated as compensatory mechanisms against mitochondrial dysfunctions.

**Figure 9:**
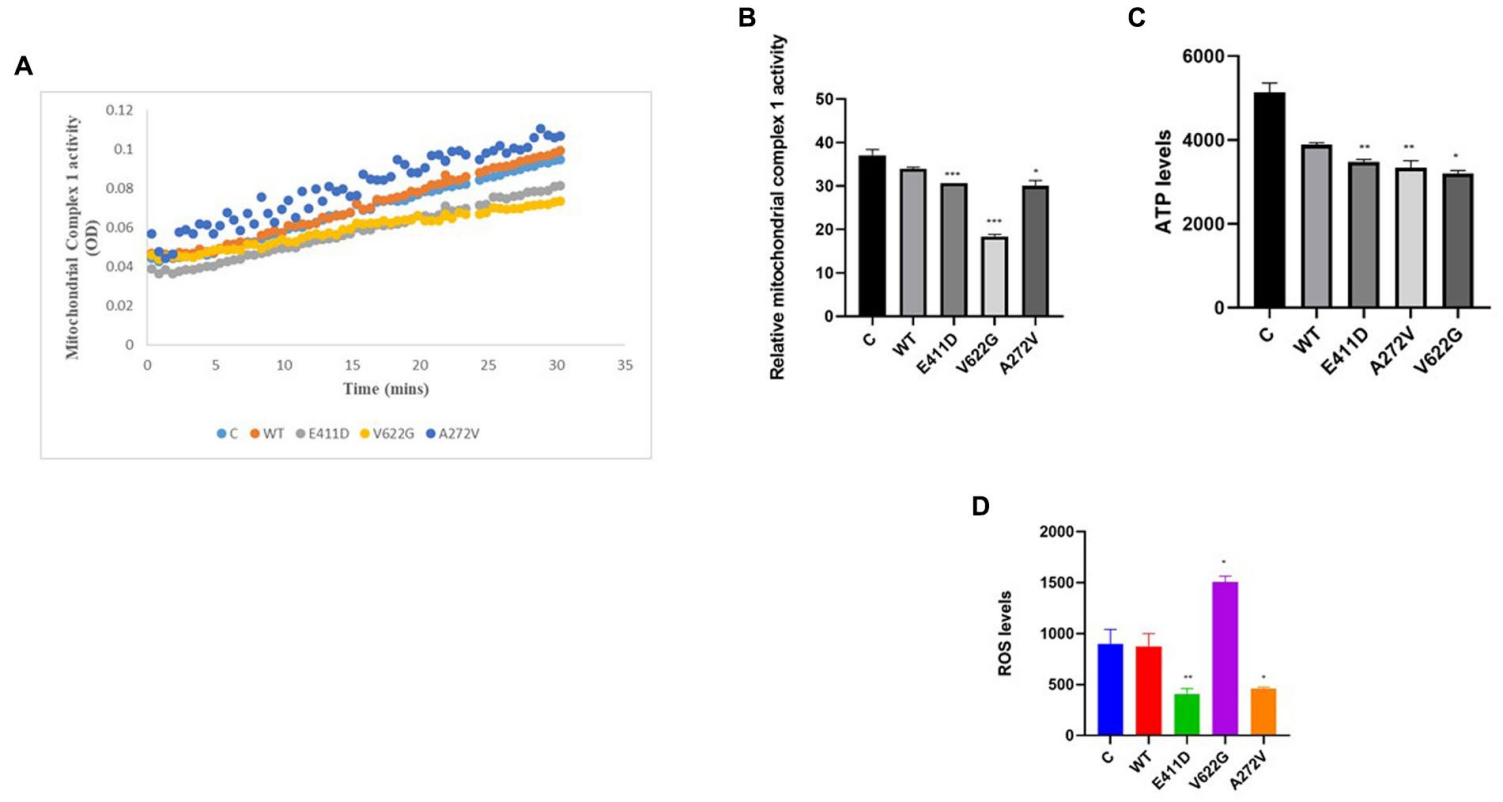
Mitochondrial complex 1 enzyme activity, ATP and ROS levels. **(A)** Line chart summarizing the mitochondrial complex 1 enzyme activity. **(B)** Histogram showing the relative mitochondrial complex 1 enzyme activity. **(C)** Histogram showing the relative ATP values. **(D)** Histogram showing the relative ROS levels. Values represent mean ± SEM of more than three independent experiments each. *p < 0.05, **p < 0.01, ***p < 0.001, ****p < 0.0001, NS, non-significant; C, negative control; and WT, wild type.

**Figure 10:**
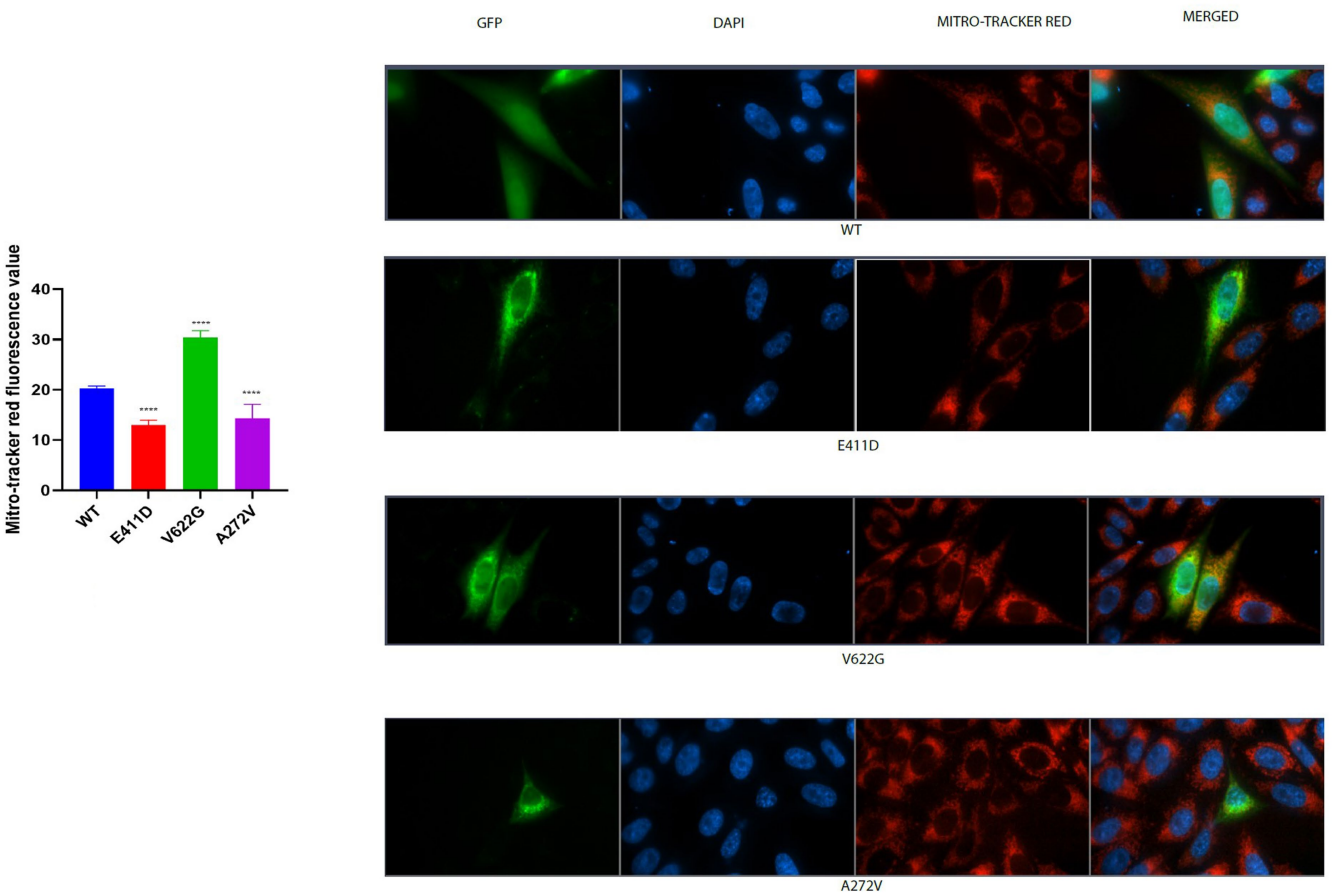
Mito-tracker red immunofluorescence intensity results. Histogram showing mitro-tracker red fluorescence values. CACNA1C protein is localized in the nucleus, cytoplasm and mitochondria. Values represent mean ± SEM of more than three independent experiments each. *p < 0.05, **p < 0.01, ***p < 0.001, ****p < 0.0001, NS, non-significant; C, negative control; and WT, wild type.

**Figure 11:**
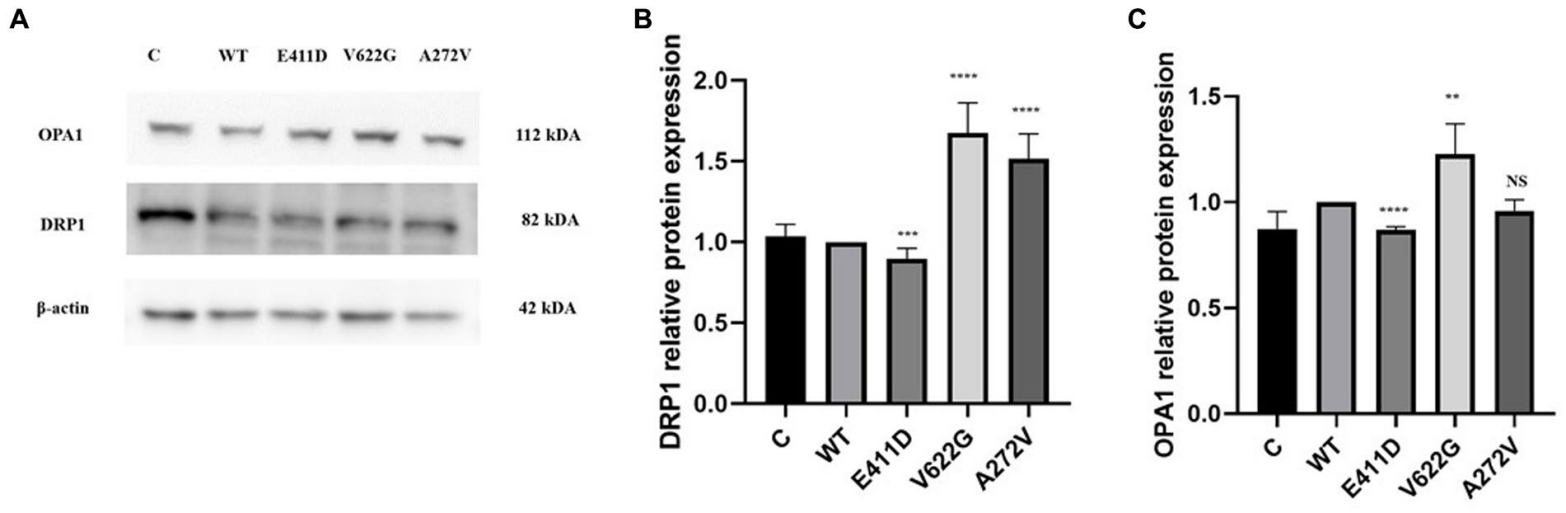
Western blot analysis for the mitochondrial fusion and fission markers. **(A)** Immunoblots of the OPA1 and DRP1 proteins. **(B)** Histogram showing the relative DRP1 protein values of respective immunoblots. **(C)** Histogram showing the relative OPA1 protein values of respective immunoblots. Values represent mean ± SEM of more than three independent experiments each. *p < 0.05, **p < 0.01, ***p < 0.001, ****p < 0.0001, NS, non-significant; C, negative control; and WT, wild type.

Mitochondrial-calcium ions homeostasis is crucial for the generation of ATP (Matuz-Mares et al., 2022). The mitochondrial complex I enzyme as a part of the oxidative phosphorylation system plays an important role in the generation of the ATP. Consequently, its impairment can alter ATP production. The highest production of ROS in the cells happens in the mitochondria (Matuz-Mares et al., 2022). Our results showed that the mitochondrial complex I enzyme activity was significantly lower than WT for all mutants (Figures 9A,B). All mutants had significantly lower ATP levels than WT (Figure 9C). The p.V622G showed considerably higher ROS levels than WT while the rest of the mutants showed significantly lower ROS levels than WT (Figure 9D). The p.E411D and p.A272V demonstrated significantly lower mito-tracker intensity when compared to WT whereas, p.V622G had significantly higher mito-tracker intensity when compared to WT (Figure 10). In addition, we could observe that CACNA1C was localized on both the nucleus and cytoplasm. Interestingly, CACNA1C was also co-localized with mitochondria (Figure 10). We also tried to check whether there was any impairment of mitochondrial fusion and fission. Mitochondrial morphology is regulated by the balance of mitochondrial fusion (facilitated by mitofusins and optic atrophy 1 (OPA1)), and fission (mediated by dynamin-related protein 1 (DRP1)). OPA1 is a protein localized in the inner mitochondrial membrane whereby it regulates mitochondrial fusion and cristae morphology, and protects cells against apoptosis. It has been shown that the DRP1 protein regulates mitochondrial dysfunction by inhibiting mitophagy, enhancing apoptosis, and increasing ROS levels (Duan et al., 2020). DRP1 protein was elevated for the p.V622G and p.A272V variants and lowered for the p.E411D variant than WT (Figures 11A,B). The p.V622G variant showed higher OPA1 protein expression than WT while p.E411D displayed lower OPA1 protein expression than WT (Figures 11A,C).

**Figure 12:**
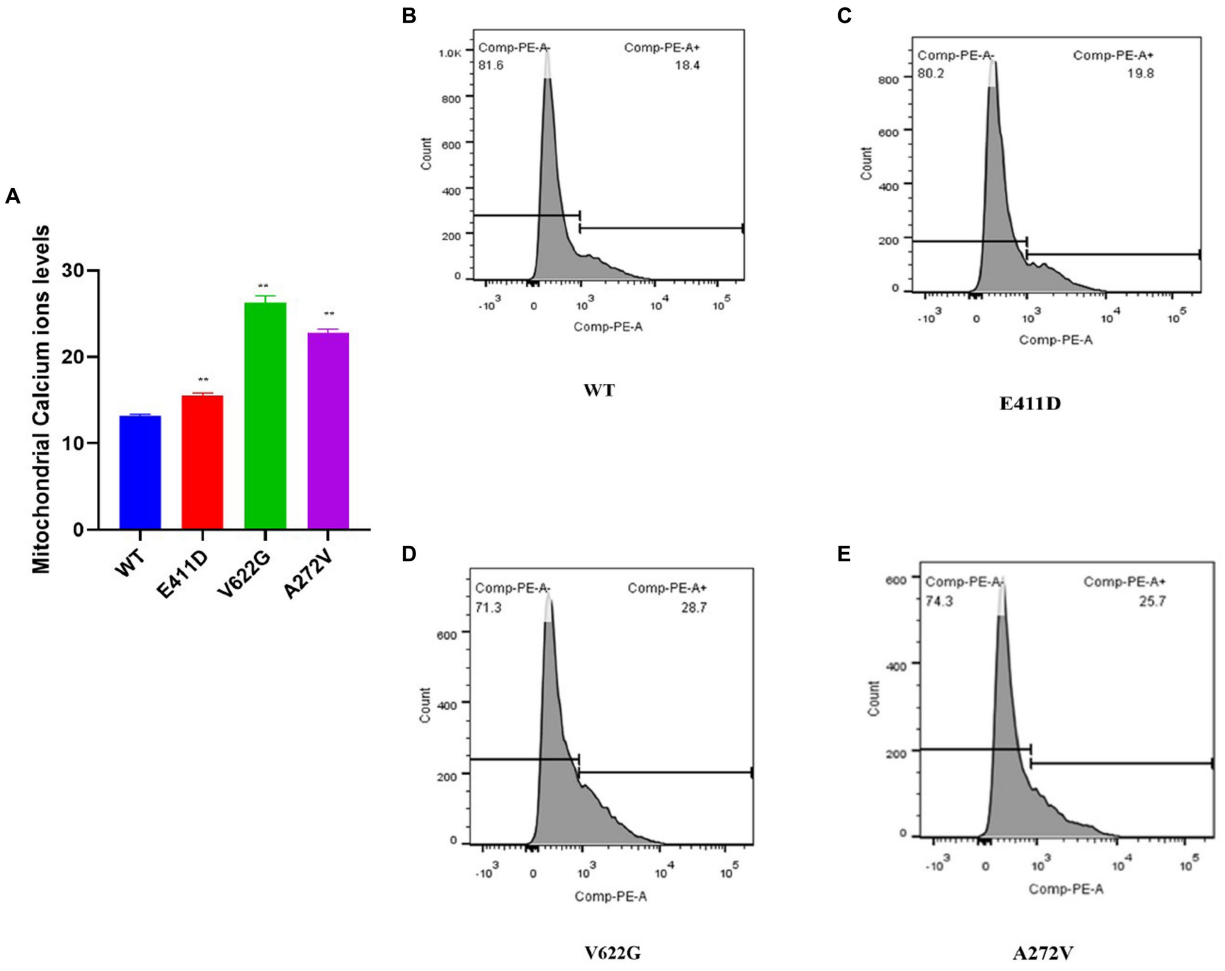
Mitochondrial calcium ions levels analysis by Rhod2-AM. **(A)** Histogram showing relative mitochondrial calcium ions levels. **(B–E)** Mitochondrial calcium ions levels for the WT and mutants. Values represent mean ± SEM of more than three independent experiments each. *p < 0.05, **p < 0.01, ***p < 0.001, ****p < 0.0001, NS, non-significant; C, negative control; and WT, wild type.

**Figure 13:**
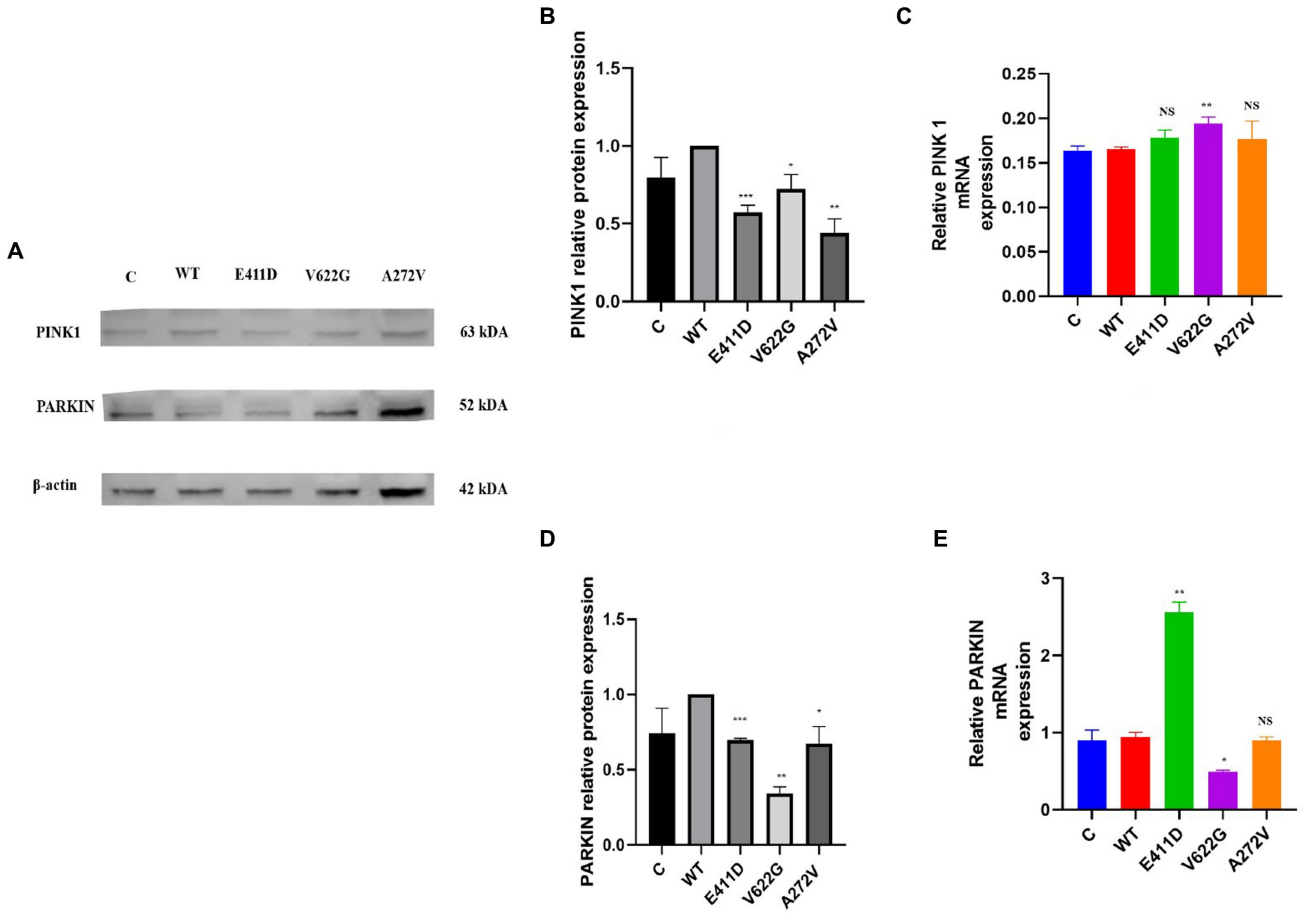
Western blot analysis and qPCR results for the mitophagy markers. **(A)** Immunoblots of the mitophagy protein markers. **(B)** Histogram showing the relative PINK1 protein values of respective immunoblots. **(C)** Histogram showing relative PINK1 mRNA expression. **(D)** Histogram showing the relative PARKIN protein values of respective immunoblots. **(E)** Histogram showing relative PARKIN mRNA expression. Values represent mean ± SEM of more than three independent experiments each. *p < 0.05, **p < 0.01, ***p < 0.001, ****p < 0.0001, NS, non-significant; C, negative control; and WT, wild type.

Mitochondrial calcium ions levels results revealed that all three mutants expressed higher levels of mitochondrial calcium ions than WT (Figures 12A–E). It has been shown that any stress condition that can result in calcium ions or ROS overload can trigger mPTP opening, causing the loss of mitochondrial membrane potential, impaired ATP production, and the release of mitochondrial proteins such as cytochrome c, which trigger cell death through apoptotic pathways (Matuz-Mares et al., 2022). Mitophagy is regulated by the PTEN Induced Kinase 1 (PINK1) and PARKIN. PINK1 and PARKIN have protective functions in the cells by regulating mitophagy and mitochondrial fission/fusion, prompting the elimination of impaired mitochondrial components, promoting mitochondrial biogenesis, and regulating the translation of mitochondrial genes (Ge et al., 2020). PINK1/PARKIN deficiency leads to mitochondrial calcium ions overload and ROS production by reducing the activity of mitochondrial sodium calcium exchanger (NCLX) and leucine zipper-EF-hand-containing transmembrane protein 1 (LETM1) (Gandhi et al., 2009; Ge et al., 2020). The p.V622G had higher PINK1 mRNA expression than WT while the rest of the mutants had non-significant changes (Figure 13C). All mutants had considerably lower PINK1 protein expression than WT (Figures 13A,B). The p.E411D expressed significantly higher PARKIN mRNA than WT while p.V622G showed significantly lower PARKIN mRNA expression than WT (Figure 13E). All mutants exhibited considerably lower PARKIN protein expression than WT (Figure 13D). Therefore, it seems like all of our mutants had lost the protective functions of mitophagy.

### Mutants displayed the presence of endoplasmic reticulum stress and impaired growth rates

**Figure 14:**
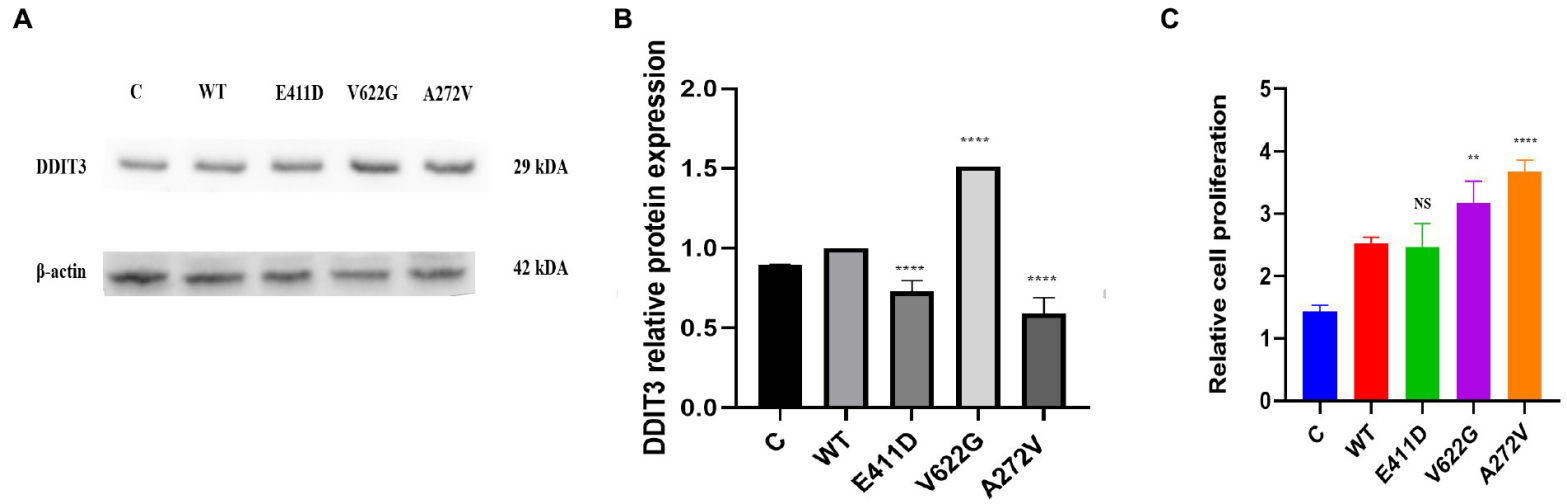
Endoplasmic reticulum stress marker and cell proliferation. **(A)** Immunoblots of the DDIT3 protein markers. **(B)** Histogram showing the relative DDIT3 protein values of respective immunoblots. **(C)** Histogram showing the relative cell proliferation rates. Values represent mean ± SEM of more than three independent experiments each. *p < 0.05, **p < 0.01, ***p < 0.001, ****p < 0.0001, NS, non-significant; C, negative control; and WT, wild type.

The p.E411D and p.A272V variants exhibited considerably lower levels of the DDIT3/CHOP protein expression than WT while p.V622G expressed significantly higher DDIT3/CHOP protein levels than WT (Figures 14A,B). The p.V622G and p.A272V variants exhibited an accelerated proliferation rate than WT while p.E411D showed no significant change (Figure 14C).

## Discussion

Similar to the previous reports (Boczek et al., 2015; Bozarth et al., 2018; Rodan et al., 2021; Herold et al., 2023), our patients presented with West syndrome, ID, ADHD, and cardiac arrhythmias. Although our mutants displayed decreased calcium current densities and reduced mRNA and protein expressions, we found that our variants could induce cell apoptosis, and affect mitochondrial functions by interfering with mitochondrial complexes, mitochondrial calcium ions influx, ATP production, ROS production, mitochondrial fusion, and fission as well as mitophagy process. The fact that our mutants exhibited reduced calcium current densities and mRNA and protein expressions, and yet exhibited elevated mitochondrial calcium ions levels, suggests that there are more unknown underlying mechanisms for the neurodevelopmental phenotypes which we have attempted to explore. The low current densities in our mutants might be due to reduced mRNA expressions that led to low CACNA1C channel protein expressions. It is unfortunate that we could not check the effect of the mutants on channel gating properties, reverse potential, and conductance in this study. However, we are looking forward to assessing them in animal models in the near future. The alternative splicing of Cavα1 pre-mRNAs is modulated differently among diverse regions of the central nervous system (Snutch et al., 1991; Diebold et al., 1992; Marubio et al., 1996; Lin et al., 1997, 1999; Welling et al., 1997; Pereverzev et al., 1998; Schramm et al., 1999; Tsunemi et al., 2002) and fluctuate according to different developmental stages (Diebold et al., 1992; Pereverzev et al., 1998). Of interest, missense, silent, and nonsense mutations have been reported to cause mRNA splicing and the missense mutations are the leading ones (Krawczak et al., 2007). It has been estimated that approximately 1.6% of missense substitutions in human genes can also affect mRNA splicing (Krawczak et al., 2007). Noteworthy, it has been shown that the regulation of the Cav1.2 channels depends on several processes such as feedback regulation, alternative splicing, and subunit composition (Herold et al., 2023). Thus, we speculate that our mutants could have affected mRNA splicing resulting in our findings. Our study coincides with previous studies that revealed the absence of a correlation between excess calcium ions influx through Cav1.2 channel and ASD (Krey et al., 2013; Li et al., 2016). Thus, some neurological deficits can result from mechanisms that are not dependent on calcium ions influx through the channel but rather in the organelles.

Calcium ions can be stored in the mitochondria, endoplasmic reticulum (Matuz-Mares et al., 2022), and lysosomes (Padamsey et al., 2019), so it seems some mutants not only affect the calcium ions influx to the cells but also the homeostasis in some organelles including the mitochondria and lysosomes. Although mitochondria play a major role in buffering cellular calcium ions, mitochondrial calcium ions overload can stimulate the opening of the mPTP resulting in the activation of apoptotic cell death (Matuz-Mares et al., 2022). Besides, it has been reported recently that presynthesized Cav1.2 channels can be found on early, recycling, and late endo/lysosomes of the cardiomyocytes (Del Villar et al., 2021; Westhoff and Dixon, 2021). Moreover, it has been revealed that the full-length C terminus of the L-type calcium channel in ventricular myocytes allocates into cytosol and nucleus (Schroder et al., 2009). This is also similar to our findings that Cav1.2 is expressed in the nucleus and cytosol, and co-localizes with both mitochondria and lysosomes. In the lysosomes, calcium ions are critical for different lysosomal functions including autophagy (Pereira et al., 2012; Medina and Ballabio, 2015; García-Rúa et al., 2016) and oxidative stress sensing (Li et al., 2012). *Cacna1c*-knockout mice died *in utero* (Seisenberger et al., 2000) indicating that homozygous LOF mutations can be lethal in human beings. Even less than a decrease of 50% in calcium current can lead to heart failure and enhance mortality in *Cacna1c*-knockout mice (Goonasekera et al., 2012). Thus, the LOF effects in our patients can explain cardiac arrhythmia.

Mitochondrial complex 1 enzyme enhances the production of ATP. Thus, its impairment can alter ATP production leading to the production of ROS (Matuz-Mares et al., 2022). High ROS levels can damage mitochondria, induce apoptosis and generate other types of complications (Hansford, 1994; Orrenius et al., 2003) including the destruction of nucleic acids (Cadet and Wagner, 2014). Our study has shown that mitochondrial complex I enzyme dysfunction can be found in *CACNA1C* variants. Similarly, one patient harboring *CACNA1C* GOF variant had partial deficits in complexes I and III based on muscle biopsy results (Garcia Segarra et al., 2014). All of our variants exhibited diminished mitochondrial complex I enzyme activity, and low ATP levels than WT. Our study has given more enlightenment that not GOF variants only can affect mitochondrial complex I enzyme activity but also LOF changes. The mitro-tracker gave more evidence that mitochondria were abnormal in comparison to WT. OPA1 protein is located in the inner mitochondrial membrane where it regulates mitochondrial fusion and cristae morphology and protects against apoptosis. The DRP1 protein regulates mitochondrial functions via the inhibition of mitophagy (it suppresses mito-Parkin recruitment), and it enhances apoptosis (it enhances the mitochondrial translocation of BAX) (Duan et al., 2020). All of our variants showed impaired mitochondrial fusion and fission and accelerated apoptosis.

We also checked several mitochondrial gene expressions and protein levels to see whether there was any dysfunction. All mutants demonstrated higher SDHA and MT-CO1 protein expression than WT suggesting the activation of the mitochondrial compensatory mechanisms. It has been shown that oxidative stress in human cells can upregulate the expression of the mitochondrial copy numbers as a feedback mechanism that compensates for defects in mitochondria carrying mutated mtDNA (Lee et al., 2000). MT-ND1 is among the mitochondrial genes that encodes for the 14 central subunits of the mitochondrial complex I enzyme (Vinothkumar et al., 2014), and is involved in the assembly of the entire mitochondrial complex I enzyme (Sharma et al., 2009). Human mitochondrial 16S rRNA gene encodes for Humanin which suppresses neuronal cell death by blocking the activation of the Bax (Guo et al., 2003), and impairment of its transportation from the mitochondria to the cytosol leads to Alzheimer’s disease (Maximov et al., 2002). Our mutants expressed higher mitochondrial copy numbers (including MT-ND1, MT3212, and 16S rRNA) than WT as compensatory mechanisms against mitochondrial dysfunction.

All of our variants exhibited cell apoptosis. The intrinsic apoptotic pathway is activated by several intracellular triggers such as oxidative stress and DNA damage (Mukhopadhyay et al., 2014). The translocation of the variants in the nucleus can be one of the causes of apoptosis. Interestingly, both qPCR and WB revealed loss of *CACNA1C* mRNA and protein expression. It has been unveiled that the deletion mutant of the C terminus of the L-type calcium channel has a greater relative affinity for the nucleus than the full-length C terminus of the L-type calcium channel, and this is consistent with increased repression of Cav1.2 promoter activity by truncated C terminus of the L-type calcium channel which in turn functions as a transcription factor regulating Cav1.2 expression (Schroder et al., 2009). Based on a fluorescence microscope, we could observe the location of the Cav1.2 channel in both the cytosol and nucleus. Therefore, we speculate that our variants might have affected the gene transcription process leading to apoptosis. Another possible explanation for the apoptosis is too much calcium ions influx to the mitochondria as shown in our results. It has been shown that PINK1/PARKIN deficiency leads to mitochondrial calcium ions overload and ROS production by reducing the activity of NCLX and LETM1 (Gandhi et al., 2009; Ge et al., 2020). Our mutants also exhibited PINK1/PARKIN deficiency. Since our mutants reduced calcium current density and CACNA1C protein expression, we think PINK1/PARKIN deficiency can explain the elevated mitochondrial calcium ions levels leading to the activation of apoptosis. In addition, calcium ions are also important for the regulation of cell proliferation (Fujita and Nakao, 1988), and some of our mutants demonstrated accelerated cell proliferation. Accelerated cell proliferation might be due to negative mechanisms against apoptosis.

Previous studies have shown that the CACNA1C channel is localized to the lysosomes and maintains the integrity of the nervous system by regulating lysosomal calcium ions homeostasis. Lysosomal calcium ions moderate lysosomal fusion and autophagy. Recently, a great improvement has been done to explain the roles of autophagy in several neurodegenerative disorders like Huntington’s disease which can somehow overlap with *CACNAIC*-related disorders (Croce and Yamamoto, 2019). There are several autophagy markers that play a role in the apoptotic pathway too, including p62 and Beclin-1 (Mukhopadhyay et al., 2014). Other autophagy markers include LC3 II and LAMP1. Most of our mutants showed the downregulation of the autophagy pathway. Almost all mutants had lower LC3II/I, Beclin-1, and LAMP-1 protein expression than WT. We speculate that our variants could have interfered with autophagy gene transcription and translation as calcium ions signaling is crucial for gene expression. Mitophagy is mitochondria-specific autophagy and is regulated by PINK1 and PARKIN. PINK1 and PARKIN have protective functions in the cells by regulating mitophagy and mitochondrial fission/fusion, prompting the elimination of impaired mitochondrial components, promoting mitochondrial biogenesis, and regulating the translation of mitochondrial genes (Ge et al., 2020). It has been shown that PINK1/PARKIN deficiency leads to mitochondrial calcium overload and ROS production by reducing the activity of NCLX and LETM1 (Gandhi et al., 2009; Ge et al., 2020). All of our mutants had PINK1/PARKIN deficiency. Although we could not explore the effects of these mutants in the endoplasmic reticulum, it is worth noting that two of our mutants expressed lower levels of the DDIT3/CHOP protein. DDIT3 also known as C/EBP homologous protein (CHOP) is a specific transcription factor in ER that can activate apoptotic pathways in many conditions such as cancer (Zhu et al., 2015). The downregulation of the DDIT3/CHOP protein might signify the inhibition of the ERS-induced apoptosis pathways as reported before in cancer (Zhu et al., 2015). Furthermore, it can imply the negative feedback mechanism against the apoptosis which affected all mutants as discussed above.

The p.E411D located on the cytoplasmic region was found in a patient with ADHD and moderate ID. This mutant exhibited a reduction of the calcium current density, low mRNA, and reduced protein expression in whole cell protein as well as in nuclear and cytoplasmic proteins. It was co-localized in the nucleus, cytoplasm, lysosome, and mitochondria. This mutant also demonstrated an accelerated apoptotic rate based on protein markers and apoptotic assay, impaired autophagy, impaired mitochondrial complex II enzyme activity according to protein change, impaired cytochrome c oxidase activity according to protein change, impaired mitochondrial copy numbers, reduced mitochondrial complex I enzyme activity, low ATP levels, impaired mitochondrial fluorescence intensity, impaired mitochondrial fusion and fission, elevated mitochondrial calcium ions, impaired mitophagy, impaired regulation of the ERS. We think the primary underlying mechanism for the ADHD and ID is probably insufficient calcium ions influx to the neurons. It has been shown that the underlying mechanisms for ASD include impaired calcium ions influx into the cell during membrane polarization for the *CACNA1C*, *CACNA1D*, *CACNA1F*, *CACNA1G*, *CACNA1I*, and *CACNA1E* genes (Smith and Walsh, 2020). Besides, it has been shown that ID can occur as a result of the calcium ions dysregulation and impaired production of ATP (Gibbs, 2015). Mitochondria supply ATP for proper brain functioning, enhancement of the synaptic plasticity, production of hormones and signaling molecules, and regulation of the neurotransmitters release (Kramer and Bressan, 2018), thus mitochondrial dysfunction might have played a role in the pathogenesis of the ID.

The p.V622G located on S4 domain II was identified in a patient who presented with West syndrome and moderate GDD. This mutant reduced calcium current density, exhibited low mRNA, and reduced protein expression in whole cell protein as well as in nuclear and cytoplasmic proteins. It was co-localized in the nucleus, cytoplasm, lysosome, and mitochondria. This mutant also demonstrated a high death rate based on protein markers and apoptotic assay, impaired autophagy, impaired mitochondrial complex II enzyme activity according to protein change, impaired cytochrome c oxidase activity according to protein change, impaired mitochondrial copy numbers, reduced mitochondrial complex I enzyme activity, low ATP levels, high ROS levels, abnormal mitochondrial fluorescence intensity, impaired mitochondrial fusion and fission, elevated mitochondrial calcium ions, impaired mitophagy, impaired regulation of the ERS and accelerated proliferation rate. Likewise, the p.A272V variant located on S5 domain I was found in a patient who presented with epilepsy and mild ID. This mutant reduced calcium current density, demonstrated low mRNA, and reduced protein expression in whole cell protein as well as in nuclear and cytoplasmic proteins too. It was co-localized in the nucleus, cytoplasm, lysosome, and mitochondria. This mutant showed an accelerated death rate based on protein markers and apoptotic assay, impaired autophagy, increased lysosomal fluorescence intensity, impaired mitochondrial complex II enzyme activity according to protein change, impaired cytochrome c oxidase activity according to protein change, impaired mitochondrial copy numbers, reduced mitochondrial complex I enzyme activity, low ATP levels, abnormal mitochondrial fluorescence intensity, impaired mitochondrial fusion and fission, elevated mitochondrial calcium ions, impaired mitophagy, impaired regulation of the ERS and accelerated proliferation rate.

The S4 helix is positively charged, therefore, it is in charge of controlling voltage-dependent activation while the loop between S5 and S6 has negatively charged residues that produce the selectivity filter (Kessi et al., 2021). Therefore, mutants located on S4, S5, and S6 can impair calcium ions influx significantly. The known mechanisms for the occurrence of epilepsy due to calcium ions dysregulation include glutamate receptor (GluR) overexcitation and changes in voltage-gated calcium channels (VGCC) activity (Wojda et al., 2008). In addition, several molecular components accountable for the maintenance of the intracellular calcium ion homeostasis, including VGCCs, endoplasmic reticulum calcium sensor stromal interaction molecule (STIM), the plasma membrane calcium channel Orai, IP3Rs and RyRs, SERCA, and transmembrane and coiled-coil domains 1 (TMCO1), have been revealed to be involved in calcium ion dysregulation that underlies epileptic seizures (Zhou et al., 2022). We speculate that insufficient calcium ions influx in the cells might have activated negative compensatory mechanisms leading to GluR over excitation as well as dysregulated intracellular calcium ions homeostasis leading to epilepsy. Low ATP levels and impaired neurotransmitter release due to insufficient calcium ions influx, might have led to the occurrence of the GDD.

## Conclusion

*CACNA1C* channels are localized in the nucleus, cytoplasm, mitochondria, and lysosomes. Both mitochondria and lysosomes are involved in the pathogenesis of *CACNA1C*-related neurodevelopmental disorders. Although our mutants had reduced current density changes according to the electrophysiological, we found that our variants could induce cell apoptosis, affect mitochondrial functions by interfering with mitochondrial complex 1 enzyme activity, mitochondrial complex II, mitochondrial cytochrome c, mitochondrial calcium ions influx, ATP production, ROS production, fusion, and fission as well as mitophagy process. We speculate that our variants impaired mRNA splicing leading to the loss of the protein expression and reduced current densities, which in turn augmented mitochondrial calcium ions influx leading to mitochondrial dysfunction, apoptosis, and mitophagy dysfunction. Therefore, we suggest researchers and scientists not rely only on the electrophysiological studies to conclude about the mechanisms of Cav1.2 channels. Future studies should focus on modifying the calcium ion change inside the organelles rather than using calcium channel blockers and openers that work on the cell membrane alone.

## Limitations

This study has some limitations that are worthy to be mentioned. There are several experiments that we could not do such as checking other mitochondrial complexes’ activities and mitochondrial potential. Besides, we could not perform transmission electron microscopy to visualize the mitochondrial morphology and we did not check the mRNA of cleaved PARP. We did not check the effect of the mutants on channel gating properties, reverse potential, and conductance. Our study was only done in cell models, therefore, animal model studies are needed to authenticate these findings. We are planning to address the above-listed limitations in animal models (in upcoming studies).

## Data availability statement

The original contributions presented in the study are included in the article/supplementary material. The original contributions presented in the study are publicly available. This data can be found in the ClinVar database: https://www.ncbi.nlm.nih.gov/clinvar/ accession numbers SCV003930352, SCV003930359 and SCV003930364.

## Ethics statement

The studies involving human participants were reviewed and approved by Institutional Ethics Committee of Xiangya Hospital, Central South University. Written informed consent to participate in this study was provided by the participants’ legal guardian/next of kin.

## Author contributions

MK and BC were the first co-authors who participated in the conception and designation of the study, performed the experiments, analyzed the data, and drafted and revised the manuscript. LP assisted in patients’ data collection and preparation of tables and some figures. LIY and LY participated in patients’ data collection. JP read and participated in the revision of the manuscript. FH and FY were the corresponding authors who participated in the conception and designation of the study, coordinated and supervised the data collection, and critically reviewed the manuscript for important intellectual content. All authors reviewed the manuscript and approved the submitted version and have agreed both to be personally accountable for the author’s own contributions and to ensure that questions related to the accuracy or integrity of any part of the work, even ones in which the author was not personally involved, are appropriately investigated, resolved, and the resolution documented in the literature.

## Funding

We are grateful for the support we received from the Hunan Natural Science Foundation (2021JJQNJJ1515) and the Natural Science Foundation of Changsha City (No. kq2208384).

## Conflict of interest

The authors declare that the research was conducted in the absence of any commercial or financial relationships that could be construed as a potential conflict of interest.

## Publisher’s note

All claims expressed in this article are solely those of the authors and do not necessarily represent those of their affiliated organizations, or those of the publisher, the editors and the reviewers. Any product that may be evaluated in this article, or claim that may be made by its manufacturer, is not guaranteed or endorsed by the publisher.
