# Supplementary material for: Disruption of mitochondrial and lysosomal functions by human CACNA1C variants expressed in HEK 293 and CHO cells
Source: Front Mol Neurosci. 2023 Jun 28;16:1209760. doi: 10.3389/fnmol.2023.1209760 (PMC10336228; doi:10.3389/fnmol.2023.1209760)
Supplement: Supplementary file 1 [file Table_1.DOCX]

**Supplementary Table 1:** List of the apoptosis, autophagy, and mitophagy primers used in this study.

| **Name of the primer** | **Primer sequence (5’- 3’)** |
| --- | --- |
| Human beta-actin-F | 5’ AGAGCTACGAGCTGCCTGAC3’ |
| Human beta-actin-R | 5’ AGCACTGTGTTGGCGTACAG 3’ |
| Human CACNA1C-F | 5’GACGTGCTGTACTGGGTCAA3’ |
| Human CACNA1C-R | 5’CCTTGTTTGCCGTGTCTTGG3’ |
| Human p62-F | 5’TGCCCAGACTACGACTTGTG3’ |
| Human p62-R | 5’AGTGTCCGTGTTTCACCTTCC3’ |
| Human LC3 II-F | 5’GAGAAGCAGCTTCCTGTTCTGG3’ |
| Human LC3 II-R | 5’GTGTCCGTTCACCAACAGGAAG3’ |
| Human Beclin-1-F | 5’GGCTGAGAGACTGGATCAGG3’ |
| Human Beclin-1-R | 5’CTGCGTCTGGGCATAACG3’ |
| Human LAMP-1-F | 5’GACAAGTACAACGTGAGCGG3’ |
| Human LAMP-1-R | 5’CGAGGTCTTGTTGGGGTTGA3’ |
| Human Caspase 3-F | 5’ATCGGACTGTGGCATTGAGA3’ |
| Human Caspase 3-R | 5’ATAACCAGGTGCTGTGGAGT3’ |
| Human Bax-F | 5’ATGGACGGGTCCGGGGAG3’ |
| Human Bax-R | 5’TCAGAAAACATGTCAGCTGCC’3 |
| Human Bcl-2-F | 5’AACTCGAGTGACAAGCCCGATG’3 |
| Human Bcl-2-R | 5’GTACCACCAGTTGGTTGTCTTTGA’3 |
| Human PARP-F | 5’GGCATCGGAACTGGACGAGG’3 |
| Human PARP-R | 5’CCCCACGAACGGAACAACCA’3 |
| Human PINK1-F | 5’TGGACACGAGACGCTTGCAG3’ |
| Human PINK1-R | 5’TCCTGGTGCACTGGTACCTG3’ |
| Human PARKIN-F | 5’GTGTTTGTCAGGTTCAACTCCA3’ |
| Human PARKIN-R | 5’GAAAATCACACGCAACTGGTC3’ |
