# Supplementary material for: Disruption of mitochondrial and lysosomal functions by human CACNA1C variants expressed in HEK 293 and CHO cells
Source: Front Mol Neurosci. 2023 Jun 28;16:1209760. doi: 10.3389/fnmol.2023.1209760 (PMC10336228; doi:10.3389/fnmol.2023.1209760)
Supplement: Supplementary file 3 [file Table_3.DOCX]

**Supplementary Table 3:** List of the mitochondrial gene primers used in this study

| **Name of the primer** | **Primer sequence (5’- 3’)** |
| --- | --- |
| Human B2M-F | 5’ TGTTCCTGCTGGGTAGCTCT3’ |
| Human B2M-R | 5’ CCTCCATGATGCTGCTTACA3’ |
| Human mt-ND1-F | 5’ CCCATGGCCAACCTCCTACTCCTC3’ |
| Human mt-ND1-R | 5’AGCCCGTAGGGGCCTACAACG3’ |
| Human hMito-1-F | 5’ CACTTTCCACACAGACATCA3’ |
| Human hMito-1-R | 5’ TGGTTAGGCTGGTGTTAGGG3’ |
| Human-16S RNA-1-F | 5’ACTTTGCAAGGAGAGCCAAA3’ |
| Human-16S RNA-1-R | 5’TGGACAACCAGCTATCACCA3’ |
| Human mt3212-F | 5’ CACCCAAGAACAGGGTTTGT3’ |
| Human mt3212-R | 5’TGGCCATGGGTATGTTGTTAA3 |
| Human beta-actin-F | 5’ AGAGCTACGAGCTGCCTGAC3’ |
| Human beta-actin-R | 5’ AGCACTGTGTTGGCGTACAG 3’ |
