# Supplementary material for: Disruption of mitochondrial and lysosomal functions by human CACNA1C variants expressed in HEK 293 and CHO cells
Source: Front Mol Neurosci. 2023 Jun 28;16:1209760. doi: 10.3389/fnmol.2023.1209760 (PMC10336228; doi:10.3389/fnmol.2023.1209760)
Supplement: Supplementary file 4 [file Table_4.DOCX]

**Supplementary Table 4**

**Genetic summary of 3 patients with CACNA1C-associated neurodevelopmental disorders in this study**

|  | **P1** | **P2** | **P3** |
| --- | --- | --- | --- |
| **Chromosome position** | Chr12-251282 | Chr12-2567764 | Chr12-2595327 |
| **Nucleic acid alteration** | c.1233G>C | c.1865T>G | c.815C>T |
| **Amino acid change** | p. E411D | p.V622G | p.A272V |
| **Transcript** | NM_000719.7 | NM_000719.6 | NM_000719 |
| **Exon / intron** | Exon 9 | Exon 13 | Exon 6 |
| **Heterozygosity** | Heterozygous | Heterozygous | Heterozygous |
| **Genetic pattern** | AD | AD | AD |
| **Mode of inheritance** | *De novo* | *De novo* | *De novo* |
| **ACMG score** | LP | VUS | LP |

**Abbreviations**: ACMG: American College of Medical Genetics, AD: autosomal dominant, P: pathogenic, LP: likely pathogenic, VUS: variant of unknown significance.
